# ADAM23 promotes neuronal differentiation of human neural progenitor cells

**DOI:** 10.1186/s11658-017-0045-1

**Published:** 2017-08-18

**Authors:** Annett Markus-Koch, Oliver Schmitt, Susanne Seemann, Jan Lukas, Dirk Koczan, Mathias Ernst, Georg Fuellen, Andreas Wree, Arndt Rolfs, Jiankai Luo

**Affiliations:** 1 Albrecht-Kossel-Institute for Neuroregeneration, Rostock University Medical Center, Gehlsheimer Straße 20, 18147 Rostock, Germany; 2 Institute of Anatomy, Rostock University Medical Center, Gertrudenstrsse 9, 18055 Rostock, Germany; 3 Institute for Immunology, Rostock University Medical Center, Schillingallee 70, 18055 Rostock, Germany; 4 Institute for Biostatistics and Informatics in Medicine and Ageing Research, Rostock University Medical Center, Ernst-Heydemann-Str. 8, 18057 Rostock, Germany

**Keywords:** ADAM23, Differentiation, Neuron, Neurite growth, hNPC

## Abstract

**Background:**

ADAM23 is widely expressed in the embryonic central nervous system and plays an important role in tissue formation.

**Results:**

In this study, we showed that ADAM23 contributes to cell survival and is involved in neuronal differentiation during the differentiation of human neural progenitor cells (hNPCs). Upregulation of ADAM23 in hNPCs was found to increase the number of neurons and the length of neurite, while its downregulation decreases them and triggers cell apoptosis. RNA microarray analysis revealed mechanistic insights into genes and pathways that may become involved in multiple cellular processes upon up- or downregulation of ADAM23.

**Conclusions:**

Our results suggest that ADAM23 regulates neuronal differentiation by triggering specific signaling pathways during hNPC differentiation.

**Electronic supplementary material:**

The online version of this article (doi:10.1186/s11658-017-0045-1) contains supplementary material, which is available to authorized users.

## Background

The members of the ADAM (a disintegrin and metalloprotease) family are type I trans-membrane proteins that have multiple functional domains, enabling them to be involved in proteolytic processes, cell adhesion and fusion, and cell signal transduction [1, 2]. ADAMs play important roles in tissue formation and morphogenesis in developing embryos [2–4] and are spatially and temporally expressed in various parts of the developing central nervous system (CNS). The major ADAMs in the developing CNS are ADAM9, 10, 17, 19, 22 and 23, which do have some overlap between them in terms of when and where they are expressed [5–11].

Some functional roles for the ADAMs in the developing CNS have been reported. For example, pharmacological inhibition of ADAM10 within a brain slice-cultured system causes the axons from retinal ganglion cells to miss their target tectum, suggesting that ADAM10 is required for correct targeting of the *Xenopus* optic projection [12]. Downregulation of ADAM10 precociously drives neuronal differentiation in the cortex and spinal cord, resulting in a severe disruption in the ventricular zone [13, 14]. ADAM17 induces gliogenesis but inhibits neurogenesis by activating epidermal growth factor receptor (EGFR) in neural stem cells [15]. Overexpression of ADAM17 induces angiogenesis by promoting vessel sprouting and increasing the number of pericytes during chicken tectal micro-vessel development [16]. In developing *Xenopus*, downregulation of ADAM19 decreases the number of neurons and neural crest cells [4].

In contrast to ADAM10 and 17, ADAM23 has no catalytic function [1, 2]. It is widely expressed in the CNS, including major parts of the brain and brain nuclei [5]. ADAM23-deficient mice display tremors and severe ataxia and die within 2 weeks of birth [17]. ADAM23 is involved in cell spreading and adherence, and synaptic transmission regulation, possibly through binding to leucine-rich glioma inactivated 1 (LGI1) and LGI4 [18]. In an embryonic carcinoma cell line of P19 cells, downregulation of ADAM23 promotes cell differentiation into neurons, with or without retinoic acid treatment, in in vitro aggregated culture, but not in monolayer cell culture [19].

ReNcell VM human neural progenitor cells (hNPCs), derived from the ventral mesencephalon of a 10-week old fetus, can differentiate in vitro into astrocytes, oligodendrocytes and neurons [20–23]. Since hNPCs are an interesting source for cell replacement in neurodegenerative disease, ReNcell VM hNPCs are a suitable model to investigate neural differentiation in humans. In this study, we assessed whether ADAM23 affects neural differentiation. Our data show that ADAM23 enhances neuronal differentiation and neurite growth in vitro during the differentiation of hNPCs.

## Methods

### Plasmid construction

The full-length sequence of chicken ADAM23, taken from previously constructed pCRII-TOPO-ADAM23 [5], was subcloned into a blunted EcoRI site of the eukaryotic expression vector pCAGGS [24] to form pCAGGS-ADAM23 (pCA-A23), as previously described [16].

Based on results obtained with the siRNA Target Finder (https://www.genscript.com/tools/sirna-target-finder), the target sequence of ggatcagattgacatcacc was chosen to downregulate endogenous human ADAM23 in ReNcell VM cells (ReNeuron). Synthetic oligonucleotides containing the siRNA target sequence were purchased from MWG (Berlin, Germany) and inserted into a hairpin siRNA-expressing vector pGSHIN2-GFP (pGS-GFP; kindly donated by Dr. Shin-Ichiro Kojima, Feinberg School of Medicine Northwestern University, USA) driven by a human H1-RNA promoter to form pGSHIN2-ADAM23 (pGS-A23). The GFP inside pGSHIN2 plasmid was used as a report gene.

### Cell culture and plasmid transfection

ReNcell VM cells were cultivated according to the previously described protocol [21]. Briefly, the cells were cultured in proliferating medium consisting of DMEM/F12 supplemented with Glutamax, B27 media supplement, heparin sodium salt and gentamycin (Invitrogen) plus 20 ng/ml epidermal growth factor (EGF; Roche) and 10 ng/ml basic fibroblast growth factor (bFGF; Roche) at 37 °C with 5% CO_2_ for 2 days. Then, plasmids for overexpression (3 μg/μl pCA-A23 in combination with 1 μg/μl pCA-GFP) or for downregulation (4 μg/μl pGS-A23 plasmid) were transfected into the proliferating cells using the Amaxa Nucleofector System (Lonza Cologne GmbH) according to the previous protocol [22]. pCA-GFP plasmid was used to label transfected cells when mixed with pCA-A23 plasmid or as a negative control when used alone. Note that the amounts of pCA-GFP and pCA-A23 plasmids were mixed with a ratio of 1:3 to ensure that the maximum amount of pCA-GFP transfected cells also expressed pCA-A23. pGS-GFP was used as a negative control for pGS-A23. Two days after transfection [designated as day 0 (d0)], cells were collected for measurement. They also continued to be triggered for differentiation by changing the differentiating medium to one that was lacking EGF and bFGF. Three (d3) or seven (d7) days after the initiation of differentiation, the cells were collected for measurement using real-time PCR, Western blot analysis and immunohistochemistry.

### Western blot analysis

Western blot analysis was performed using the previously described protocol [20]. The primary human ADAM23 antibody (Santa Cruz) is a rabbit polyclonal antibody raised against amino acids 351–415 (etwtekdqidittnpvqmlhefskyrqrikqhadavhlisrvtfhykrsslsyfggvcsrtrgvgand) within an extracellular domain of human ADAM23 (NP_003803). The primary chicken ADAM23 antibody (generated by a custom service from Eurogentec) is a rabbit polyclonal antibody against amino acids asmqlqdhetesssew and cirdtgnkkdegpkgb of chicken ADAM23 (NP-001138702.1) [25].

Note that the epitope sequences of the antibodies against human and chicken ADAM23 were not compatible, so there was no cross-reaction between the species in the Western blot detection. GAPDH (Abcam) and the secondary antibodies of Alexa Fluor 680 from goat anti-rabbit or anti-mouse, and of goat anti-mouse IRDye 800 (all from Invitrogen) were used. GAPDH was used as a loading control for the Western blot. Protein visualization and quantification were performed with an Odyssey Infrared Imaging System (LI-COR Biosciences GmbH) according to the manufacturer’s instructions.

### Cell survival assay

The cultivated and manipulated cells were collected and washed with PBS. The viability of living cells was measured using the CASY Technology System (Innovatis) according to the manufacturer’s instructions.

### Cell apoptosis assay

The number of apoptotic cells in the cultured slices was evaluated using the TdT-mediated dUTP nick end labeling (TUNEL) assay using the In Situ Cell Death Detection Kit - TMR red (Roche) according to the manufacturer’s instructions. The slices were imaged using a fluorescent microscope (BZ-8000; Keyence Deutschland GmbH) and the fluorescence-positive cells were counted using the Keyence software.

### Flow cytometry

Cells were harvested by treatmemt with 2.5 ml trypsin (Gibco) and 5 ml tritin (Sigma), both supplemented with 25 units/ml of benzonase (Merck). Collected cells were fixed for 15 min with 1% formaldehyde solution in PBS, the antibody (Tuj-1; Santa Cruz) raised against βIII-tubulin in Saponin buffer (0.5% Saponin, 0.5% BSA, 0.02% NaN_3_ in PBS) was added, and the cells were incubated for 2 h at room temperature. The Alexa-Fluor 647-labeled secondary antibody (Moleculcar Probes) was added after the cells were washed with PBS solution. Finally, the samples were measured using a FACS Calibur (BD Bioscience).

### Immunocytochemistry

ReNcell VM cells were proliferated in chamber slices coated with laminin and subsequently differentiated as described above. After the cells had been incubated for 30 min with blocking solution (5% normal goat serum with 0.3% TritonX-100 in PBS), the mouse primary antibody Tuj-1 (Santa Cruz) was added and incubated for 30 min at room temperature. After a wash with PBS, the secondary antibodies Alexa Fluor 488 or Alexa Fluor 568 goat anti-mouse (Molecular Probes) were added. Incubation was for 60 min at room temperature. Subsequently, the slides were embedded with mounting medium containing DAPI (Vectashield) and the staining was imaged using a fluorescent microscope system (BZ-8000; Keyence Deutschland GmbH).

### Measurement of neurite length

The neurite length of βIII-tubulin-positive cells in the slices was quantified by counting the intersection of fibers with a grid of cycloids (cycloid length: 30 μm) according to a previously described stereological analytical method [26]. The length density L(A) was calculated with the formula: L(A) = 2 x (ΣQ(A))/(a(frame) x ΣP(ref)), where ΣQ(A) is the total number of intersections of fibers with the cycloids; ΣP(ref) is the total number of points associated with the test frame that fall within the reference area, and a(frame) is the area of the test frame [26]. Only the fibers that possess an ending connecting to a DAPI-positive nucleus were counted. Stereo Investigator v8.0 (Micro-Bright Field) with an Olympus BX-51 light microscope and MBF Bioscience CX9000 camera were used to define sampling areas and evaluate the cell culture frames.

The neurite length of βIII-tubulin positive cells in slices was also quantified using the Keyence BZ-8000 microscope with Keyence software according to the manufacturer’s instructions. Only the fibers extending from DAPI-positive nuclei were analyzed. For each condition, at least 80 to 100 cells were measured.

### Oligonucleotide microarray

Cells were transfected with pCA-A23 for overexpression or pGS-A23 for downregulation of ADAM23, and with pCA-GFP or pGS-GFP as controls. They were harvested and prepared for transcriptome analysis 3 days after the initiation of differentiation.

The Affymetrix GeneChip Human 1.0 ST Array system was used to perform the RNA microarray according to the manufacturer’s instructions. The so-called whole transcriptome (WT) protocol starts with first-strand synthesis by introducing T7 promoter tags to all RNA molecules using N6 3’ends. After strand replacement according to Eberwine, non-labeled aRNA is produced via in vitro transcription in concert with the linear amplification of all RNA molecules without a 3’bias. After cleanup of the aRNA, a new, identical single-strand DNA is produced using the aRNA as template by adding random primers and dNTPs to replace a certain amount of dTTP by dUTP. After cleanup, including RNaseH digestion, an endpoint fragmentation can be performed, using uracyldeglycosidase to remove the uracil in combination with apurinic apyrimidinic endonuclease 1 (APE1), cleaving the deuracylized phosphodiester backbone of the single strand DNA. A desoxynucleotidyltransferase is used to add biotinylated dNTPs to the 3’ends of the fragmented single strand DNA fragments. The hybridisation was carried out overnight at 45 °C in a GeneChip Hybridisation Oven 640 (Affymetrix). The microarray was scanned using the GeneChip Scanner 3000 (Affymetrix) at 0.7 μm resolution.

The microarray data was analyzed with the Partek Genomics Suite 6.5 software using the robust multichip analysis (RMA) algorithm including an adjustment to the GC content (GCRMA). An analysis of variance (ANOVA) was performed to contrast the candidate genes between the groups of three biological replicates. The quality control and the production of gene expression data was performed by using the Affymetrix software. Analyses of differentially expressed genes and gene ontology (GO) enrichment were carried out using R software version 3.2.2.

### Statistical analysis

Results are represented as means ± SEM from at least three independent experiments. The statistical evaluation was carried out with the two-tailed Student’s t-test using Excel software (Microsoft). A difference was considered to be significant when the *p*-value was less than 0.05 (*p* < 0.05).

## Results

### ADAM23 is essential for cell survival

**Fig. 1:**
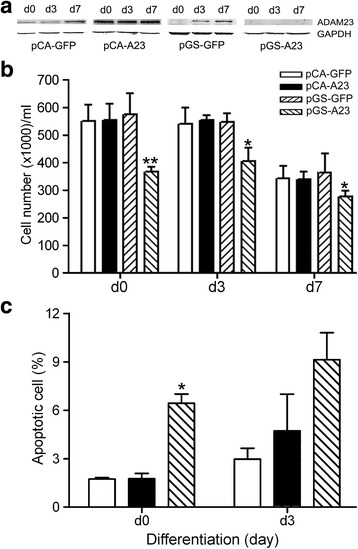
Cell survival and apoptosis analyses after up- and downregulation of ADAM23 in ReNcell VM cells. **a** Western blot analyses of: endogenous ADAM23 in the control groups with pCA-GFP or pGS-GFP plasmid-transfected cells revealed by human ADAM23 antibody (lanes in pCA-GFP and pGS-GFP); upregulated ectopic ADAM23 in pCA-A23-transfected cells revealed by chicken ADAM23 (lanes in pCA-A23); and downregulated endogenous ADAM23 in pGS-A23-transfected cells (lanes in pGS-A23) revealed by human ADAM23 antibody. The images were taken on proliferation day (d0), and 3 (d3) or 7 days (d7) after the initiation of differentiation. GAPDH was used as a loading control. **b** The cell viability in the pGS-A23-transfected group decreased when compared to the control pGS-A23 and pCA-A23 groups on proliferation day (d0) and 3 (d3) and 7 days (d7) after the initiation of differentiation, as revealed with the CASY assay. pCA-GFP and pGS-GFP were used as controls. Data are presented as mean ± SEM from at least three independent experiments. *p < 0.05 and **p < 0.01. **c** The percentages of apoptotic cells were measured 2 days after transfection with pCA-A23 and pGS-A23 plasmids on d0 and 3 days (d3) after the initiation of differentiation. pCA-GFP was used as a control. Data are presented as mean ± SEM from at least three independent experiments. *p < 0.05

ReNcell VM cells were transfected with pCA-A23 (co-transfected with pCA-GFP) and with pGS-A23 shRNA plasmids to respectively up- or downregulate ADAM23. The cells were collected at proliferation state (d0), and 3 (d3) or 7 days (d7) after the initiation of differentiation. The FACS analysis showed that the percentages of green fluorescent protein-positive (GFP-positive) cells 2 days after transfection were about 50% in different groups of controls (pCA-GFP and pGS-GFP), suggesting that the plasmids were successfully transfected into the cells (data not shown). The Western blot analysis demonstrated that endogenous ADAM23 protein gradually increased as the cells differentiated (lanes in pCA-GFP and pGS-GFP; Fig. 1a), and ectopic ADAM23 was showed increased expression in the transfected cells (lanes in pCA-A23; Fig. 1a). Endogenous ADAM23 was downregulated in the pGS-A23-transfected cells (lanes in pGS-A23; Fig. 1a) on d3 and d7, suggesting that the plasmids worked well in the ReNcell VM cells.

Next, we investigated whether up- or downregulation of ADAM23 affected the survival of cultured living cells. The data revealed that the number of cultured living cells in the pGS-A23-transfected group decreased when compared to the control (pGS-GFP) and pCA-A23-transfected groups (Fig. 1b), suggesting that downregulation of ADAM23 in cells affected cell survival. Note that the cell number of the controls, pCA-GFP and pGS-GFP, was also lower on d7 compared to that on d0 and d3 (Fig. 1b), suggesting that some cells underwent a cell death process by d7, regardless of handling.

We also investigated cell apoptosis using the TUNEL assay and demonstrated that downregulation of ADAM23 increased the number of apoptotic cells on d0. There was a similar tendency for d3 (Fig. 1c).

### ADAM23 regulates neuronal differentiation

**Fig. 2:**
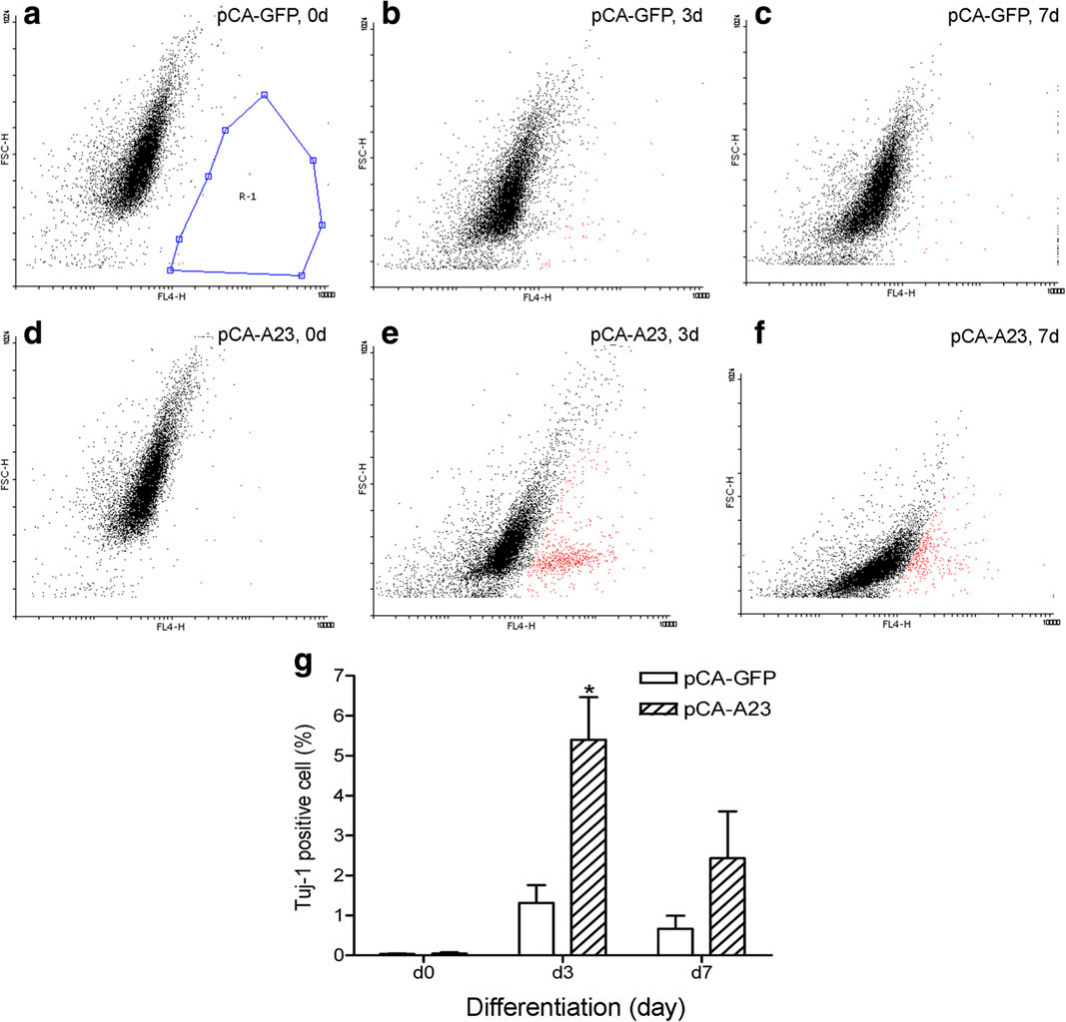
Upregulation of ADAM23 increases neuronal differentiation, as shown by flow cytometry measurements. The dot plots with a forward scatter (FSC-H) vs. fluorescence (FL4-H) show the total cell and fluorescence-positive cell numbers after transfection with pCA-GFP control (**a**–**c**) and pCA-A23 (**d**–**f**) 3 days (d3) and 7 days (d7) after the initiation of differentiation. The percentages of Tuj-1-positive cells were also measured (**g**). Note that the Tuj-1-positive cells were marked as *red* in the dot plots, as indicated by R-1 region. Overexpression of pCA-A23 obviously increased the number of red dots. Data are presented as mean ± SEM from at least three independent experiments. *p < 0.05

**Fig. 3:**
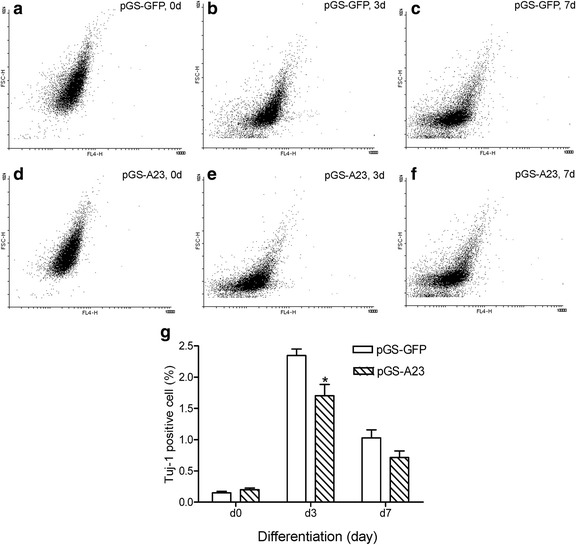
Downregulation of ADAM23 decreases neuronal differentiation, as shown by flow cytometry measurements. The dot plots with a forward scatter (FSC-H) vs. fluorescence (FL4-H) show the total cell and fluorescence-positive cell numbers after transfection with pGS-GFP control (**a**–**c**) and pGS-A23 (**d**–**f**) 3 days (d3) and 7 days (d7) after the initiation of differentiation. The percentages of Tuj-1 positive cells were also measured (**g**). Note that the Tuj-1-positive cells were marked as *red* in the dot plots. Data are presented as mean ± SEM from at least three independent experiments. *p < 0.05

To investigate the effect of ADAM23 on neural differentiation, transfected cells underwent immunocytochemistry staining at differentiation stages with the antibody Tuj-1 against the neuronal marker βIII-tubulin. This was followed by a flow cytometry analysis. βIII-tubulin is widely used as a neuronal marker in studies on the differentiation of ReNcell VM cells [20–23]. The dot plot analyses showed that the percentage of Tuj-1-positive cells (red dots) significantly increased in the pCA-A23-transfected cells (Fig. 2), but decreased in pGS-A23-transfected cells (Fig. 3) compared to the controls on d3, suggesting that ADAM23 is involved in neurite growth during the differentiation of hNPCs.

### ADAM23 regulates neurite growth

**Fig. 4:**
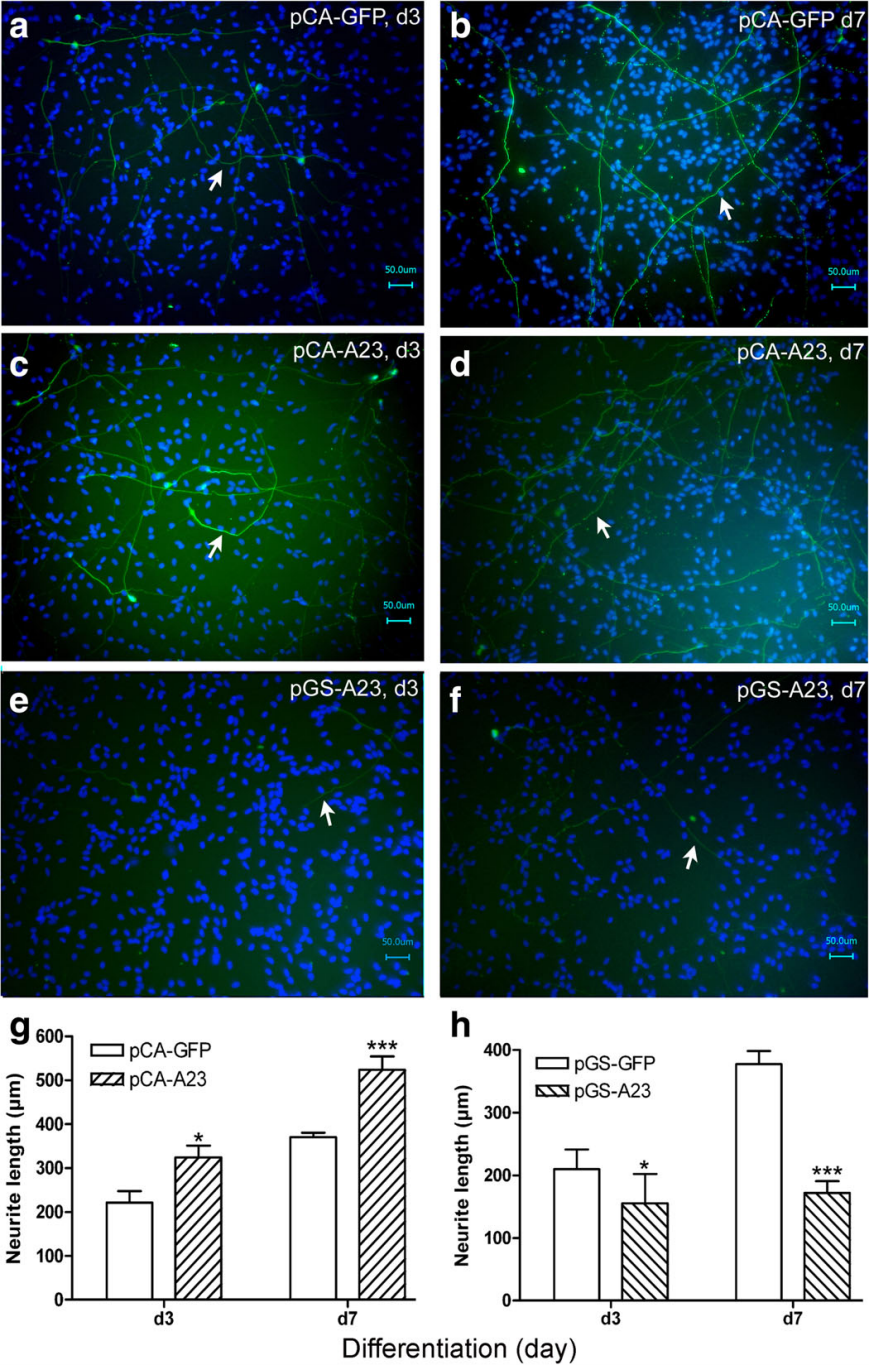
The effects of ADAM23 on the neurite length, as measured via stereological analysis. **a**–**f** Immunohistochemistry images of cells transfected with pCA-GFP (**a**, **b**), pCA-A23 (**c**, **d**), and pGS-A23 (**e**, **f**), showing the neurite length of Tuj-1-positive cells 3 days (d3; **a**, **c**, **e**) and 7 days (d7; **b**, **d**, **f**) after the initiation of differentiation. *Arrows* indicate the Tuj-1-positive neurites (*green*). Cell nuclei are colored *blue*. Scale bar: 50 μm for all. **g**, **h** Quantification of the neurite length measured using a stereological analytical method after transfection with pCA-A23 (**g**) or pGS-A23 (**h**). The measurements were taken on d3 and d7 after the initiation of differentiation. Data are presented as mean ± SEM from at least three independent experiments. *p < 0.05 and ***p < 0.001

After ADAM23 was found to be respectively up- or downregulated by pCA-A23 or pGS-A23 transfection, immunocytochemistry was performed using Tuj-1 antibody. Stereological analysis was applied to the immunocytochemistry images to measure the neurite length of Tuj-1-positive cells. The results showed that upregulation of ADAM23 significantly increased the neurite length of the Tuj-1-positive cells on d3 and d7 (Fig. 4g), while its downregulation significantly decreased the length (Fig. 4h) when compared to the controls, pCA-GFP and pGS-GFP (Fig. 4g, h). These results were also obtained when the neurite length was directly measured in the Tuj-1-positive images using Keyence BZ-Analyzer Software, with results comparable to those from the stereological analysis (data not shown).

### ADAM23 impacts on transcriptional expression

**Fig. 5:**
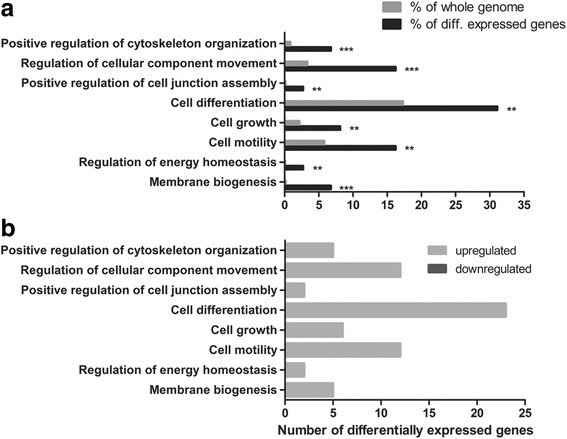
GO enrichment of differentially expressed genes after overexpression of ADAM23. **a** Significantly enriched GO terms related to differentially expressed genes after overexpression of ADAM23 on differentiation day 3. *Grey* columns indicate the percentage of the GO term in the whole genome, while *black* columns represent the subset of differentially expressed genes. **p < 0.01 and *** *p* < 0.001. **b** The ratio of up- and downregulated genes in the subset of differentially expressed genes bearing the same GO term

To elucidate possible mechanisms for ADAM23 effects on cell survival and neuronal differentiation, RNA microarray analysis was performed in triplicate on ADAM23 overexpressing and underexpressing cells 3 days after the initiation of differentiation. The results showed 74 differentially regulated genes in ADAM23 overexpressing cells compared to the control (Additional file 1: Table S1A). GO enrichment analysis of these genes revealed 122 distinct GO terms with a significantly higher abundance than expected (Additional file 2: Table S2A). These GO terms included cell differentiation, growth and motility (Fig. 5a). The differentially expressed genes annotated with these GO terms are exclusively upregulated, suggesting that they may be involved in the enhanced neuronal differentiation and neurite outgrowth observed with ADAM23 overexpression. Additional file 3: Table S3A shows the differentially expressed genes. Double enrichment of differentially expressed genes after the overexpression of ADAM23 revealed 4 genes (ACTA2, CTGF, CYR61 and ANXA2) with a more than twofold change in gene expression. These are also annotated with the selected GO terms shown in Fig. 5.

**Fig. 6:**
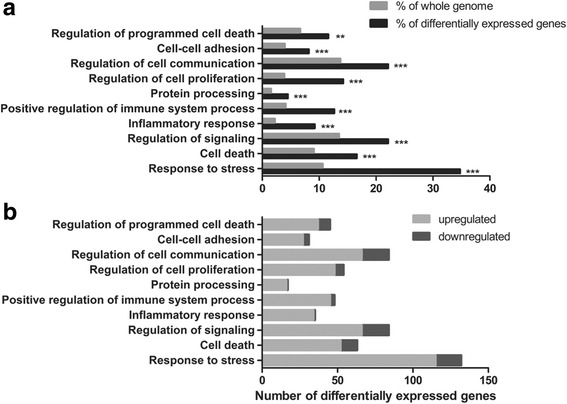
GO enrichment of differentially expressed genes after downregulation of ADAM23. **a** Significantly enriched GO related to differentially expressed genes after knockdown of ADAM23 on differentiation day 3. The *grey* column indicates the percentage of the GO term in the whole genome, while the *black* column represents the subset of differentially expressed genes. **p < 0.01 and ***p < 0.001. **b** The ratio of up- and downregulated genes in the subset of differentially expressed genes bearing the same GO term

In downregulated ADAM23 cells, 380 differentially expressed genes were identified (Additional file 4: Table S1B) and found to be related to 335 distinct GO terms exhibiting a higher abundance relative to the whole genome (Additional file 5: Table S2B). The GO terms included cell death and response to stress. Other terms refer to more specific processes triggered by them (Fig. 6a), suggesting that downregulation of ADAM23 compromised the generation and survival of neurons in the culture. Additional file 6: Table S3B shows the differentially expressed genes. Double enrichment of differentially expressed genes after the downregulation of ADAM23 revealed 29 genes with a more than threefold change in gene expression. These also carry the selected GO terms shown in Fig. 6.

The GO terms mentioned in Fig. 5 were used to create a GO tree (Additional file 7: Fig. S1), indicating all significantly enriched GO terms after overexpression of ADAM23 (yellow circles) and required connective GO terms (red circles). The lower part of the tree indicates lower generation, i.e., less specific GO terms, whereas the terms become more specific approaching the top. Therefore, each further generation more specifically describes the impact of ADAM23 overexpression in the ReNcell VM cell line.

## Discussion

Our data suggest that ADAM23 contributes to cell survival and is involved in neuronal differentiation by directly or indirectly regulating specific targeting genes that remain to be defined.

The ADAM23 gene is conserved in human, monkey, mouse, rat and chicken. For example, chicken ADAM23 protein (NP_001138702.1) shows 80% sequence identity to human ADAM23 (NP_003803). Previous data have indicated that ADAM23 can bind to its ligand in different orthologues. For example, LGI1 protein secreted from HEK293T cells via its conserved common binding site binds to chicken and rat ADAM23 protein of cultured neurons isolated from chicken dorsal root ganglia and rat hippocampus and induces neurite outgrowth [27, 28]. In our experiments, overexpression of chicken ADAM23 protein in hNPCs (Fig. 1) promoted neuronal differentiation (Figs. 2, 3 and 4) and altered different genes (Figs. 5 and 6).

ADAM23 is expressed widely in the developing brain [5, 25], the cochlea and the retina [9, 11]. During late development of the chicken embryo, different regions of the chicken brain show various expression patterns of the ADAM23 protein [25]. For example, the amount of ADAM23 shows a gradual increase from embryonic day (E) 10, reaches a peak on about E14, and then decreases until E20 in the telencephalon, tectum and hindbrain; but it stays stable in the diencephalon, and gradually increases to a peak at E20 in the cerebellum [25]. This suggests that ADAM23 regulates brain development in a specifically regional-dependent manner.

LGIs are specific binding partners for ADAM22 and ADAM23, and the interaction between LGIs and ADAMs regulates distinct cell responses in different cell types [18, 29]. ADAM23 is not essential for normal neuronal cell migration, but required for the regulation of neurite outgrowth via binding with LGI1 [28, 30, 31]. Mutation of ADAM23 in mice alters dendritic arborization and brain circuitry, lowers seizure thresholds, and induces seizure activity in individuals with autosomal dominant partial epilepsy with auditory features [30], suggesting an important role for ADAM23 in CNS development.

Downregulation of ADAM23 drives embryonic carcinoma P19 cells to differentiate into neuroectodermal cells [19], where ADAM23 suppresses neuronal differentiation via its disintegrin domain by inhibiting P27KIP1 function [32]. ADAM23 can also bind to αvβ3 integrin and negatively modulate the function of αvβ3 activation, resulting in tumor metastasis [33]. Here, we showed that the expression of ADAM23 in differentiating hNPCs gradually increases to a high level on day 7 (Fig. 1), indicating a functional effect of ADAM23 on cell differentiation. Furthermore, upregulation of ADAM23 in hNPCs in monolayer cell culture still promotes neuronal differentiation (Figs. 2 and 4), unlike with P19 cells [19], suggesting that the mechanisms of ADAM23 regulating neuronal differentiation in distinct cell types are different, possibly mediated by binding to different ligands according to cell types and the cellular micro-environment. Thus, ADAM23 interacts with different ligands and receptors in a cell type-specific manner and triggers different signaling pathways, resulting in, e.g., tumor metastasis, cell proliferation and differentiation, but also in the suppression of neuronal fate.

Using RNA microarray we identified that up- and downregulation of ADAM23 respectively induce an increase or decrease of specific categories of genes during hNPC differentiation (Figs. 5 and 6), suggesting that ADAM23 drives neuronal differentiation by directly or indirectly affecting target genes. For example, the mRNA level of meteorin (METRNL), which is primarily responsible for cell differentiation and negative regulation of the inflammatory response, is upregulated about 1.5-fold in ADAM23-overexpressing cells compared to the control group (Additional file 1: Table S1A). The mRNA amount of TNF-related apoptosis-inducing ligand (TNFSF10 or TRAIL), which is connected with cell apoptotic and necroptotic processes [34], is promoted about sevenfold in ADAM23 downregulated cells compared to the control (Additional file 4: Table S1B). The precise mechanisms of how ADAM23 triggers different signaling pathways should be further investigated.

## Conclusion

During hNPC differentiation, overexpression of ADAM23 induces neurogenesis and neurite growth, whereas its downregulation triggers cell apoptosis and inhibition of neuronal differentiation. This suggests that ADAM23 is involved in neuronal differentiation, triggering specific signaling pathways that remain to be elucidated.

## Additional files

Additional file 1: Table S1A. Differentially expressed genes after overexpression of ADAM23. (PDF 344 kb)

Additional file 2: Table S2A. Enriched GO terms after overexpression of ADAM23. (PDF 213 kb)

Additional file 3: Table S3A. GO selection after overexpression of ADAM23 and included genes. (PDF 201 kb)

Additional file 4: Table S1B. Differentially expressed genes after downregulation of ADAM23. (PDF 392 kb)

Additional file 5: Table S2B. Enriched GO terms after downregulation of ADAM23. (PDF 271 kb)

Additional file 6: Table S3B. GO selection after downregulation of ADAM23 and included genes. (PDF 245 kb)

Additional file 7: Fig. S1. The GO tree for significantly enriched GO terms after overexpression of ADAM23. Significantly enriched GO terms (yellow circles) after overexpression of ADAM23 are illustrated in a GO tree together with the required connective GO terms (red circles). All GO terms are represented together with their corresponding daughter GO terms. Twelve generations of GO terms are included. The GO term at the bottom presents the first-generation GO term (molecular function). (PDF 87 kb)

## Abbreviations

ADAM: A disintegrin and metalloprotease
CNS: Central nervous system
EGF: Epidermal growth factor
EGFR: Epidermal growth factor receptor
GO: Gene ontology
hNPCs: Human neural progenitor cells
LGI1: Leucine-rich glioma inactivated 1
TUNEL: TdT-mediated dUTP nick end labeling
