# Supplementary material for: ADAM23 promotes neuronal differentiation of human neural progenitor cells
Source: Cell Mol Biol Lett. 2017 Aug 18;22:16. doi: 10.1186/s11658-017-0045-1 (PMC5562998; doi:10.1186/s11658-017-0045-1)
Supplement: Supplementary file 1 — Differentially expressed genes after overexpression of ADAM23. (PDF 344 kb) [file 11658_2017_45_MOESM1_ESM.pdf]

Differentially expressed genes after overexpression of ADAM23

p-values < 0.05

GeneSymbol	RefSeq	FoldChange
A2M	NM_000014	1,9157
ACTA2	NM_001141945	3,2951
ACTN1	NM_001130004	1,6215
ADCY8	NM_001115	-1,6067
ALK	NM_004304	1,7352
ANXA1	NM_000700	1,6341
ANXA2	NM_001002858	2,0003
ANXA2P2	NR_003573	1,8045
APOL4	NM_030643	1,8941
BCL6	NM_001706	1,5425
C11orf88	NM_207430	1,5498
C21orf62	NM_001162495	1,9159
C21orf63	NM_058187	2,4027
CAV1	NM_001753	1,8375
CCBE1	NM_133459	1,5238
CCDC39	NM_181426	1,6510
CDCA2	NM_152562	1,5950
CHODL	NM_024944	1,7207
CNN2	NM_004368	1,5428
CP	NM_000096	1,7753
CPNE4	NM_130808	1,8909
CRY1	NM_004075	1,8218
CTGF	NM_001901	2,1800
CYP4F12	NM_023944	-1,5974
CYR61	NM_001554	2,2676
DDR2	AY423733	1,5164
DEPDC1	NM_001114120	1,5289
DOCK5	NM_024940	1,5638
ELMOD1	NM_018712	1,8506
EMP1	NM_001423	1,9597
EMP2	NM_001424	1,5131
ESCO2	NM_001017420	1,5900
FAM183A	NM_001101376	1,5434
FBXO32	NM_058229	1,6256
FHL2	NM_201555	1,5455
FREM1	NM_144966	-2,1225
FRMD3	NM_174938	1,8616
GABRQ	NM_018558	3,1562
GJA1	NM_000165	1,5314
GLIPR1	NM_006851	3,5069
GRIK1	NM_175611	1,5777
KCNJ13	NM_002242	2,9354
LEPREL1	NM_018192	2,2745
LOC643923	ENST00000299326	1,6518
LUM	NM_002345	1,5505

MAPK4	NM_002747	1,5714
MBOAT1	NM_001080480	1,5123
METRNL	NM_001004431	1,5359
MFSD2	NM_001136493	1,8137
NEDD9	NM_001142393	1,6729
NPAS4	NM_178864	1,9319
NR4A3	NM_173198	1,5555
PCDH8	NM_002590	-1,5130
PMAIP1	NM_021127	1,5304
PODN	NM_153703	1,9122
PODXL	NM_001018111	1,9429
PSRC1	NM_001032290	1,7125
RAB27B	NM_004163	-1,6848
RFTN1	NM_015150	1,9391
ROPN1L	NM_031916	1,5086
S100A10	NM_002966	1,8068
S100A11	NM_005620	1,8010
SHROOM3	NM_020859	1,6817
SLN	NM_003063	2,6037
SNORD25	NR_002565	-1,5777
SNTG1	NM_018967	1,6385
STOML3	NM_145286	1,6993
TAGLN	NM_001001522	1,5815
TFPI2	NM_006528	2,4402
TMEM132E	NM_207313	2,2633
TPM1	NM_000366	1,5035
WBSCR17	NM_022479	1,7223
WDR63	NM_145172	1,5032
ZNF385D	NM_024697	1,5991
