# Supplementary material for: ADAM23 promotes neuronal differentiation of human neural progenitor cells
Source: Cell Mol Biol Lett. 2017 Aug 18;22:16. doi: 10.1186/s11658-017-0045-1 (PMC5562998; doi:10.1186/s11658-017-0045-1)
Supplement: Supplementary file 2 — Enriched GO terms after overexpression of ADAM23. (PDF 213 kb) [file 11658_2017_45_MOESM2_ESM.pdf]

Significantly enriched GO terms after overexpression of ADAM23

	GOBPID	Pvalue	Count	Size	Term
1	GO:0001765	2,57E-10	5	10	membrane raft assembly
2	GO:0044091	1,62E-07	5	31	membrane biogenesis
3	GO:0051270	1,55E-05	12	667	regulation of cellular component movement
4	GO:0048646	2,20E-05	15	1067	anatomical structure formation involved in morphogenesis
5	GO:0051336	5,75E-05	15	1159	regulation of hydrolase activity
6	GO:0061572	0.000107702278234067	5	114	actin filament bundle organization
7	GO:0003071	0.000128941610978739	3	24	renal system process involved in regulation of systemic arterial blood pressure
8	GO:0031340	0.000167648467112967	2	5	positive regulation of vesicle fusion
9	GO:0022610	0.000257481227715581	15	1325	biological adhesion
10	GO:0030199	0.000515676563018481	3	38	collagen fibril organization
11	GO:0090257	0.000547123562134964	3	41	regulation of muscle system process
12	GO:0051496	0.000557005382676497	3	39	positive regulation of stress fiber assembly
13	GO:0008217	0.00059851349417766	5	165	regulation of blood pressure
14	GO:0051495	0.000615153476765236	5	166	positive regulation of cytoskeleton organization
15	GO:0048729	0.000656267520464731	9	587	tissue morphogenesis
16	GO:0032060	0.000744399008611646	2	10	bleb assembly
17	GO:1903044	0.000744399008611646	2	10	protein localization to membrane raft
18	GO:0072359	0.000749507038237721	11	863	circulatory system development
19	GO:0043062	0.000766091963365605	7	366	extracellular structure organization
20	GO:0071384	0.000965258895053984	3	47	cellular response to corticosteroid stimulus
21	GO:0009887	0.00104882091300148	11	899	organ morphogenesis
22	GO:0060429	0.00106198015104636	12	1046	epithelium development
23	GO:0003093	0.00108596797534628	2	12	regulation of glomerular filtration
24	GO:0016264	0.00108596797534628	2	12	gap junction assembly
25	GO:0065009	0.00116644617137025	21	2584	regulation of molecular function
26	GO:0031116	0.00127999446753306	2	13	positive regulation of microtubule polymerization
27	GO:0045669	0.00144645272060681	3	54	positive regulation of osteoblast differentiation
28	GO:0035988	0.00148934607130751	2	14	chondrocyte proliferation
29	GO:2000505	0.00148934607130751	2	14	regulation of energy homeostasis
30	GO:0051179	0.00151997184799696	33	5108	localization
31	GO:0003013	0.00156544414081217	7	414	circulatory system process
32	GO:0043085	0.00167469639027664	14	1419	positive regulation of catalytic activity
33	GO:0032970	0.00172162836641723	6	309	regulation of actin filament-based process
34	GO:0048518	0.00174821923148933	32	4924	positive regulation of biological process
35	GO:0051247	0.00191271092322533	14	1439	positive regulation of protein metabolic process
36	GO:0060080	0.00195352047674901	2	16	inhibitory postsynaptic potential
37	GO:0050878	0.00207357141940598	9	692	regulation of body fluid levels
38	GO:0001944	0.00213739583758463	8	562	vasculature development
39	GO:0042060	0.00219895505650804	9	698	wound healing
40	GO:0043331	0.00257607173293135	3	66	response to dsRNA
41	GO:0030155	0.00259899883336974	8	580	regulation of cell adhesion
42	GO:0046903	0.00265489928302195	11	1010	secretion
43	GO:0048870	0.00269226423615897	12	1167	cell motility
44	GO:0031032	0.00269253455896767	4	140	actomyosin structure organization
45	GO:0044699	0.00275948684110024	59	12320	single-organism process
46	GO:0003351	0.00276158926964407	2	19	epithelial cilium movement
47	GO:0097205	0.00276158926964407	2	19	renal filtration
48	GO:0071260	0.00292368338210469	3	69	cellular response to mechanical stimulus
49	GO:0051384	0.00320982627772394	4	147	response to glucocorticoid
50	GO:0032231	0.00329886904148293	3	72	regulation of actin filament bundle assembly
51	GO:0048514	0.00331044149442486	7	473	blood vessel morphogenesis
52	GO:0061082	0.0037008735006732	2	22	myeloid leukocyte cytokine production
53	GO:0086064	0.0037008735006732	2	22	cell communication by electrical coupling involved in cardiac conduction
54	GO:0006937	0.00379124312425379	4	154	regulation of muscle contraction
55	GO:2000106	0.00384317771747391	3	76	regulation of leukocyte apoptotic process
56	GO:0051130	0.00386504783067797	10	937	positive regulation of cellular component organization
57	GO:0010952	0.00387975013476216	4	155	positive regulation of peptidase activity
58	GO:1903034	0.00388769667458817	6	364	regulation of response to wounding
59	GO:0033043	0.00399243524050742	10	911	regulation of organelle organization
60	GO:0055117	0.00413464168692706	3	78	regulation of cardiac muscle contraction
61	GO:0003065	0.00414438502673797	1	1	positive regulation of heart rate by epinephrine
62	GO:0009444	0.00414438502673797	1	1	pyruvate oxidation
63	GO:0010232	0.00414438502673797	1	1	vascular transport
64	GO:0033031	0.00414438502673797	1	1	positive regulation of neutrophil apoptotic process

65	GO:0038097	0.00414438502673797	1	1	positive regulation of mast cell activation by Fc-epsilon receptor signaling pathway
66	GO:0060156	0.00414438502673797	1	1	milk ejection
67	GO:0097350	0.00414438502673797	1	1	neutrophil clearance
68	GO:1900085	0.00414438502673797	1	1	negative regulation of peptidyl-tyrosine autophosphorylation
69	GO:0048145	0.00428528011890318	3	79	regulation of fibroblast proliferation
70	GO:1901890	0.00439834427445255	2	24	positive regulation of cell junction assembly
71	GO:0031099	0.00484314353526718	4	165	regeneration
72	GO:0007229	0.00508838863399889	3	84	integrin-mediated signaling pathway
73	GO:0051053	0.00508838863399889	3	84	negative regulation of DNA metabolic process
74	GO:0031401	0.00523572829965561	11	1104	positive regulation of protein modification process
75	GO:0001952	0.00525913609222571	3	85	regulation of cell-matrix adhesion
76	GO:0040012	0.00542457137785003	8	655	regulation of locomotion
77	GO:0030029	0.00550531703574408	5	288	actin filament-based process
78	GO:0060306	0.00554908794053412	2	27	regulation of membrane repolarization
79	GO:0048545	0.00605346340410573	6	399	response to steroid hormone
80	GO:0051899	0.00616455428823859	3	90	membrane depolarization
81	GO:0010517	0.00674978850240493	3	93	regulation of phospholipase activity
82	GO:0010165	0.00682246242262243	2	30	response to X-ray
83	GO:0045923	0.00682246242262243	2	30	positive regulation of fatty acid metabolic process
84	GO:0043065	0.00698408474300562	7	543	positive regulation of apoptotic process
85	GO:1902580	0.00708055690197697	11	1150	single-organism cellular localization
86	GO:0010811	0.00715771561954736	3	95	positive regulation of cell-substrate adhesion
87	GO:0086002	0.00727363207456834	2	31	cardiac muscle cell action potential involved in contraction
88	GO:0007160	0.00770699661565246	3	102	cell-matrix adhesion
89	GO:0001937	0.0077379670114882	2	32	negative regulation of endothelial cell proliferation
90	GO:0045823	0.0077379670114882	2	32	positive regulation of heart contraction
91	GO:0045907	0.0077379670114882	2	32	positive regulation of vasoconstriction
92	GO:0051385	0.0077379670114882	2	32	response to mineralocorticoid
93	GO:0001869	0.00827187002762997	1	2	negative regulation of complement activation, lectin pathway
94	GO:0002041	0.00827187002762997	1	2	intussusceptive angiogenesis
95	GO:0003294	0.00827187002762997	1	2	atrial ventricular junction remodeling
96	GO:0009785	0.00827187002762997	1	2	blue light signaling pathway
97	GO:0010260	0.00827187002762997	1	2	organ senescence
98	GO:0010652	0.00827187002762997	1	2	positive regulation of cell communication by chemical coupling
99	GO:0014839	0.00827187002762997	1	2	myoblast migration involved in skeletal muscle regeneration
100	GO:0032764	0.00827187002762997	1	2	negative regulation of mast cell cytokine production
101	GO:0032804	0.00827187002762997	1	2	negative regulation of low-density lipoprotein particle receptor catabolic process
102	GO:0036292	0.00827187002762997	1	2	DNA rewinding
103	GO:0038063	0.00827187002762997	1	2	collagen-activated tyrosine kinase receptor signaling pathway
104	GO:0071386	0.00827187002762997	1	2	cellular response to corticosterone stimulus
105	GO:0086098	0.00827187002762997	1	2	angiotensin-activated signaling pathway involved in heart process
106	GO:1901492	0.00827187002762997	1	2	positive regulation of lymphangiogenesis
107	GO:1901877	0.00827187002762997	1	2	negative regulation of calcium ion binding
108	GO:2000304	0.00827187002762997	1	2	positive regulation of ceramide biosynthetic process
109	GO:2000832	0.00827187002762997	1	2	negative regulation of steroid hormone secretion
110	GO:2000850	0.00827187002762997	1	2	negative regulation of glucocorticoid secretion
111	GO:0043434	0.00829182624009364	7	561	response to peptide hormone
112	GO:0030334	0.00836944968661176	7	562	regulation of cell migration
113	GO:0003206	0.00846813333547439	3	101	cardiac chamber morphogenesis
114	GO:0051290	0.00870568046723402	2	34	protein heterotetramerization
115	GO:0016049	0.00870880769197475	6	431	cell growth
116	GO:1902589	0.00916810226851354	17	2268	single-organism organelle organization
117	GO:0003091	0.00920883456102291	2	35	renal water homeostasis
118	GO:0035850	0.00920883456102291	2	35	epithelial cell differentiation involved in kidney development
119	GO:0048871	0.00937536364762903	5	312	multicellular organismal homeostasis
120	GO:0072659	0.00945354249704703	4	200	protein localization to plasma membrane
121	GO:0010942	0.00968607878078512	7	578	positive regulation of cell death
122	GO:0030154	0.00999826166455713	23	3485	cell differentiation
