# Supplementary material for: ADAM23 promotes neuronal differentiation of human neural progenitor cells
Source: Cell Mol Biol Lett. 2017 Aug 18;22:16. doi: 10.1186/s11658-017-0045-1 (PMC5562998; doi:10.1186/s11658-017-0045-1)
Supplement: Supplementary file 3 — GO selection after overexpression of ADAM23 and included genes. (PDF 201 kb) [file 11658_2017_45_MOESM3_ESM.pdf]

GO selection for differentially expressed genes after overexpression of ADAM23

GO ID	GO term	Count in OV cells	Percentage of counts in OV cells [%]	Count in all genes	Percentage of counts in all genes [%]	p value	Significance
GO:0044091	membrane biogenesis	5	6,76	31	0,15	1,62E-07	***
GO:2000505	regulation of energy homeostasis	2	2,70	14	0,07	0,0015	**
GO:0048870	cell motility	12	16,22	1167	5,79	0,0027	**
GO:0016049	cell growth	6	8,11	431	2,14	0,0087	**
GO:0030154	cell differentiation	23	31,08	3485	17,30	0,0100	**
GO:1901890	positive regulation of cell junction assembly	2	2,70	24	0,12	0,0044	**
GO:0051270	regulation of cellular component movement	12	16,22	667	3,31	1,55E-05	***
GO:0051495	positive regulation of cytoskeleton organization	5	6,76	166	0,82	0,0006	***

GO ID	GO term	Counts	Gene (Unterschiedlich exprimiert(+/-xfach))																						
GO:0044091	membrane biogenesis	5	EMP2	S100A10	CAV1	RFTN1	ANXA2																		
		5 up	+ 1.51309	+ 1.80679	+ 1.83746	+ 1,93914	+ 2.00027																		
GO:2000505	regulation of energy homeostasis	2	METRNL	NR4A3																					
		2 up	+ 1.53588	+ 1.55553																					
GO:0048870	cell motility	12	TPM1	EMP2	DDR2	CCBE1	GJA1	ANXA1	CCDC39	CAV1	PODN	PODXL	CTGF	CYR61											
		12 up	+1.50349	+ 1.51309	+ 1.51641	+ 1.52379	+ 1.53136	+ 1.63411	+ 1.651	+ 1.83746	+ 1.91218	+ 1.94286	+ 2.18002	+ 2.26759											
GO:0016049	cell growth	6	GJA1	BCL6	PSRC1	EMP1	CTGF	CYR61																	
		6 up	+ 1.53136	+ 1.54245	+ 1.71253	+ 1.95968	+ 2.18002	+ 2.26759																	
GO:0030154	cell differentiation	23	TPM1	EMP2	DDR2	GJA1	METRNL	BCL6	FHL2	NR4A3	TAGLN	ESCO2	ACTN1	ANXA1	SHROOM3	CHODL	ALK	S100A10	CAV1	A2M	PODXL	ANXA2	CTGF	CYR61	ACTA2
		23 up	+1.50349	+ 1.51309	+ 1.51641	+ 1.53136	+ 1.53588	+ 1.54245	+ 1.54545	+ 1.55553	+ 1.58147	+ 1.58997	+ 1.62147	+ 1.63411	+ 1.6817	+ 1.7207	+ 1.73524	+ 1.80679	+ 1.83746	+ 1.91572	+ 1.94286	+ 2.00027	+ 2.18002	+ 2.26759	+ 3.29507
GO:1901890	positive regulation of cell junction assembly	2	S100A10	CAV1																					
		2 up	+ 1.80679	+ 1.83746																					
GO:0051270	regulation of cellular component movement	12	TPM1	EMP2	DDR2	CCBE1	GJA1	BCL6	ACTN1	CCDC39	CAV1	PODN	PODXL	CYR61											
		12 up	+1.50349	+ 1.51309	+ 1.51641	+ 1.52379	+ 1.53136	+ 1.54245	+ 1.62147	+ 1.651	+ 1.83746	+ 1.91218	+ 1.94286	+ 2.26759											
GO:0051495	positive regulation of cytoskeleton organization	5	TPM1	PSRC1	S100A10	CAV1	CTGF																		
		5 up	+1.50349	+ 1.71253	+ 1.80679	+ 1.83746	+ 2.18002																		
