# Supplementary material for: ADAM23 promotes neuronal differentiation of human neural progenitor cells
Source: Cell Mol Biol Lett. 2017 Aug 18;22:16. doi: 10.1186/s11658-017-0045-1 (PMC5562998; doi:10.1186/s11658-017-0045-1)
Supplement: Supplementary file 4 — Differentially expressed genes after downregulation of ADAM23. (PDF 392 kb) [file 11658_2017_45_MOESM4_ESM.pdf]

**Differentially expressed genes after knockdown of ADAM23**

p-values < 0.05

| GeneSymbol | RefSeq | FoldChange |
|---|---|---|
| A2ML1 | NM_144670 | -1,5024 |
| ABCC3 | NM_003786 | 1,7354 |
| ABCC4 | NM_005845 | -1,8165 |
| ABCD2 | NM_005164 | -1,5295 |
| ACO1 | NM_002197 | -1,5564 |
| ACSL5 | NM_016234 | 1,5628 |
| ACTN1 | NM_001130004 | 1,5695 |
| ACTN2 | NM_001103 | -1,7146 |
| ADAMTS14 | NM_139155 | 1,5986 |
| ADAMTS15 | NM_139055 | 1,8281 |
| ADCY8 | NM_001115 | -1,9096 |
| AK3L1 | NM_001005353 | -1,7586 |
| ALDOC | NM_005165 | -1,8224 |
| ALK | NM_004304 | 1,6697 |
| ALPK2 | NM_052947 | 1,9870 |
| AMIGO2 | NM_001143668 | -1,5547 |
| ANKFN1 | NM_153228 | -1,9045 |
| ANKH | NM_054027 | -1,5825 |
| ANKRD20A1 | NM_032250 | -1,5176 |
| ANKRD20A3 | NM_001012419 | -1,5071 |
| ANKRD20B | NR_003366 | -1,6316 |
| ANXA1 | NM_000700 | 1,5416 |
| ANXA2 | NM_001002858 | 1,9492 |
| APOBEC3B | NM_004900 | 1,5077 |
| APOBEC3F | NM_145298 | 1,8002 |
| APOBEC3G | NM_021822 | 2,6753 |
| APOL1 | NM_145343 | 2,3756 |
| APOL2 | NM_030882 | 1,6461 |
| APOL4 | NM_030643 | 2,2108 |
| APOL6 | NM_030641 | 6,0366 |
| AQP1 | NM_198098 | -1,6798 |
| ARHGAP29 | NM_004815 | 2,0802 |
| ARHGAP6 | NM_013427 | -1,5963 |
| ASPHD2 | NM_020437 | 1,7032 |
| ASS1 | NM_000050 | 1,7141 |
| ATP10A | NM_024490 | 2,2194 |
| ATP1A2 | NM_000702 | -1,8947 |
| B3GAT2 | NM_080742 | -1,9369 |
| BATF2 | NM_138456 | 2,3226 |
| BRCA2 | NM_000059 | 1,5937 |
| BTBD11 | NM_001018072 | 1,9802 |
| BTN3A3 | NM_006994 | 2,7226 |
| C10orf81 | AK292867 | 1,5288 |
| C14orf159 | NM_001102366 | 1,6833 |
| C14orf83 | BC104940 | 1,9305 |

| | | |
|---|---|---|
| C1orf158 | BC029894 | -1,5028 |
| C1orf95 | NM_001003665 | -1,7267 |
| C1R | NM_001733 | 2,4322 |
| C1S | NM_201442 | 3,0599 |
| C21orf63 | NM_058187 | 2,3444 |
| C3 | NM_000064 | 10,9911 |
| C5orf56 | AK096941 | 2,0553 |
| C7orf41 | NM_152793 | -1,7045 |
| CA13 | NM_198584 | 1,7789 |
| CA2 | NM_000067 | 1,7852 |
| CABP7 | NM_182527 | 1,8274 |
| CACNG5 | NM_145811 | 2,9527 |
| CARD16 | NM_052889 | 2,1471 |
| CASP1 | NM_033292 | 5,1311 |
| CASZ1 | NM_001079843 | 1,6812 |
| CAV1 | NM_001753 | 1,8596 |
| CD274 | NM_014143 | 2,0980 |
| CD36 | NM_001001548 | -2,5803 |
| CD74 | NM_001025159 | 4,8605 |
| CD9 | NM_001769 | 1,5905 |
| CDC25B | NM_021873 | 1,7030 |
| CDCA2 | NM_152562 | 1,6311 |
| CDH7 | NM_033646 | -1,5465 |
| CEBPB | NM_005194 | 1,6585 |
| CEBPD | NM_005195 | 1,8305 |
| CEP135 | NM_025009 | 1,7900 |
| CFB | NM_001710 | 4,1060 |
| CFI | NM_000204 | 2,0330 |
| CHI3L1 | NM_001276 | 2,2838 |
| CHODL | NM_024944 | 1,7439 |
| CHRM3 | NM_000740 | 1,8408 |
| CLDN1 | NM_021101 | 5,2334 |
| CMPK2 | NM_207315 | 4,5018 |
| CMTM4 | NM_181521 | -1,5383 |
| CNGA4 | NM_001037329 | -1,5054 |
| CNR1 | NM_016083 | -1,5898 |
| COL4A1 | NM_001845 | 1,5134 |
| CP | NM_000096 | 1,7855 |
| CPNE5 | NM_020939 | -1,5269 |
| CRY1 | NM_004075 | 1,6772 |
| CSAG2 | NM_001080848 | 3,2452 |
| CSF1 | NM_000757 | 1,6819 |
| CSGALNACT1 | NM_018371 | -2,1846 |
| CSMD1 | NM_033225 | -1,6754 |
| CSMD3 | NM_198124 | -1,8130 |
| CTGF | NM_001901 | 2,0401 |
| CTSH | NM_004390 | 1,5266 |
| CTSO | NM_001334 | 1,6371 |
| CXCL10 | NM_001565 | 15,7926 |
| CXCL11 | NM_005409 | 2,9439 |

| | | |
|---|---|---|
| CXCL16 | NM_022059 | 2,3651 |
| CYP21A2 | NM_000500 | 2,1142 |
| CYP4F12 | NM_023944 | -1,8625 |
| CYP7B1 | NM_004820 | -1,5123 |
| CYR61 | NM_001554 | 2,3858 |
| DDR2 | NM_001014796 | 1,8650 |
| DHX58 | NM_024119 | 3,3855 |
| DNAH12 | NM_178504 | -1,5857 |
| DPP4 | NM_001935 | 1,6761 |
| DRAM1 | NM_018370 | 1,6317 |
| DRD4 | NM_000797 | 1,5176 |
| EFCAB4B | NM_001144958 | 2,3497 |
| EHD4 | NM_139265 | 1,8569 |
| ELFN1 | NM_001128636 | 1,7416 |
| ELMO1 | NM_014800 | -1,8237 |
| ELMOD1 | NM_018712 | 1,8460 |
| ELN | NM_000501 | 1,8456 |
| EMP2 | NM_001424 | 1,5313 |
| ENPP2 | NM_006209 | 2,3045 |
| EPB41L4B | NM_019114 | -2,3677 |
| EPSTI1 | NM_001002264 | 1,7857 |
| ERAP2 | NM_022350 | 2,3876 |
| ETV5 | NM_004454 | 2,0842 |
| ETV6 | NM_001987 | 1,5399 |
| ETV7 | NM_016135 | 1,5757 |
| FAM107A | NM_007177 | 1,5329 |
| FAM46A | NM_017633 | 1,5890 |
| FAM65B | NM_014722 | 1,6631 |
| FAM65C | AK299337 | 1,7109 |
| FAM95B1 | AL833349 | -1,6214 |
| FAP | NM_004460 | -2,3142 |
| FAR2 | NM_018099 | 2,6867 |
| FBLN2 | NM_001004019 | 1,5193 |
| FBLN5 | NM_006329 | 2,5019 |
| FBXO39 | NM_153230 | 4,4204 |
| FLJ42875 | NR_015440 | -1,5330 |
| FLVCR2 | NM_017791 | 1,5134 |
| FOS | NM_005252 | 2,0710 |
| FOSL2 | NM_005253 | 2,3864 |
| FREM1 | NM_144966 | -2,6492 |
| FTSJD2 | NM_015050 | 1,5018 |
| FZD5 | NM_003468 | 1,6973 |
| GABRG3 | NM_033223 | 1,5249 |
| GABRQ | NM_018558 | 4,7078 |
| GALNT13 | NM_052917 | -1,5542 |
| GBE1 | NM_000158 | -1,5689 |
| GBP1 | NM_002053 | 11,3596 |
| GBP3 | NM_018284 | 5,1533 |
| GBP4 | NM_052941 | 1,9021 |
| GCNT4 | NM_016591 | 1,7765 |

| | | |
|---|---|---|
| GJA1 | NM_000165 | 1,7643 |
| GLIPR1 | NM_006851 | 3,1559 |
| GLRX | NM_002064 | 1,7207 |
| GNG4 | NM_001098721 | -1,6495 |
| GPR155 | NM_001033045 | -1,9724 |
| GRAMD1B | NM_020716 | -1,6415 |
| GRB10 | NM_001001555 | 1,5090 |
| GRB14 | NM_004490 | -1,6941 |
| GULP1 | NM_016315 | -1,7366 |
| H19 | NR_002196 | 1,5516 |
| H1F0 | NM_005318 | 1,6805 |
| HAS2 | NM_005328 | 2,5118 |
| hCG_2009921 | AK056484 | -1,5201 |
| HCP5 | NM_006674 | 4,0385 |
| HERC5 | NM_016323 | 7,7781 |
| HIST1H2BD | NM_021063 | 1,9008 |
| HIST1H2BK | NM_080593 | 1,7155 |
| HIST2H2BF | NM_001024599 | 1,7606 |
| HLA-DMA | NM_006120 | 2,5088 |
| HLA-DPA1 | NM_033554 | 5,6366 |
| HLA-DPB1 | NM_002121 | 2,0214 |
| HLA-DQB1 | NM_002123 | 1,5225 |
| HLA-DRB5 | NM_002125 | 2,6518 |
| HLA-F | NM_018950 | 2,8629 |
| | NM_001098479 | |
| HMP19 | NM_015980 | -1,7793 |
| HNF4G | NM_004133 | 1,5265 |
| ICAM1 | NM_000201 | 3,1168 |
| IER3 | NM_003897 | 1,5471 |
| IFI16 | NM_005531 | 1,7573 |
| IFI27 | NM_001130080 | 7,0215 |
| IFI30 | NM_006332 | 4,6927 |
| IFIT2 | NM_001547 | 4,3777 |
| IFIT5 | NM_012420 | 2,3822 |
| IFITM2 | NM_006435 | 2,2499 |
| IL13RA1 | NM_001560 | 1,7104 |
| IL34 | NM_152456 | 1,5020 |
| IRAK2 | NM_001570 | 1,6189 |
| IRF1 | NM_002198 | 2,6956 |
| IRF7 | NM_004031 | 1,9384 |
| IRS1 | NM_005544 | 1,5121 |
| ITGA3 | NM_002204 | 2,3470 |
| ITGA5 | NM_002205 | 1,6268 |
| JUNB | NM_002229 | 1,8322 |
| KCND3 | NM_004980 | 1,9892 |
| KCNIP1 | NM_001034837 | 1,8011 |
| KCNK2 | NM_001017425 | -1,6624 |
| KIAA0746 | NM_015187 | 2,4568 |
| KIAA1618 | NM_020954 | 2,1581 |
| LAMP3 | NM_014398 | 1,6335 |

| | | |
|---|---|---|
| LBA1 | NM_014831 | 2,7161 |
| LEFTY2 | NM_003240 | 1,5890 |
| LGALS9 | NM_009587 | 1,8260 |
| LGALS9B | NM_001042685 | 1,8571 |
| LGALS9C | NM_001040078 | 1,5936 |
| LOC100131112 | ENST00000438484 | -1,5891 |
| LOC643763 | NR_027378 | -3,1595 |
| LOC643923 | ENST00000299326 | 1,7779 |
| LOXL2 | NM_002318 | 1,5376 |
| LOXL4 | NM_032211 | 1,8069 |
| LPAR3 | NM_012152 | -1,6800 |
| LRRC1 | NM_018214 | -1,5836 |
| MAF | NM_001031804 | 1,5320 |
| MAN1C1 | NM_020379 | 1,6198 |
| MAOB | NM_000898 | 2,0706 |
| MAPK4 | NM_002747 | 1,7809 |
| MEF2C | NM_002397 | 1,7060 |
| MFSD2 | NM_001136493 | 1,7927 |
| MMP19 | NM_002429 | 1,6290 |
| MOV10 | NM_020963 | 1,5956 |
| MPPED2 | NM_001584 | -1,6622 |
| MSTN | NM_005259 | -3,0757 |
| MT1DP | NR_027781 | 1,6834 |
| MT1F | NM_005949 | 2,1361 |
| MT1G | NM_005950 | 2,6025 |
| MT1JP | AF348994 | 1,6194 |
| MT1M | NM_176870 | 1,7779 |
| MT1X | NM_005952 | 1,7153 |
| MT2A | NM_005953 | 2,6769 |
| MX2 | NM_002463 | 4,9764 |
| MYH9 | NM_002473 | 2,0466 |
| MYO5B | NM_001080467 | 1,6412 |
| NCRNA00152 | NR_024204 | 2,0262 |
| NEBL | NM_006393 | -1,7183 |
| NEDD9 | NM_001142393 | 1,7250 |
| NEUROG2 | NM_024019 | -1,6729 |
| NFKBIZ | NM_031419 | 2,0762 |
| NIPAL2 | NM_024759 | 1,7817 |
| NKAIN3 | NM_173688 | -1,8653 |
| NLRC5 | NM_032206 | 3,4024 |
| NMI | NM_004688 | 4,0555 |
| NNMT | NM_006169 | 2,0001 |
| NOD2 | NM_022162 | 1,8550 |
| NUAK2 | NM_030952 | 1,5864 |
| OASL | NM_003733 | 5,4445 |
| OLFML2B | NM_015441 | 1,9094 |
| OPRK1 | NM_000912 | -1,7111 |
| OPTN | NM_001008211 | 1,6117 |
| OR2B6 | NM_012367 | 2,3439 |
| OR52H1 | NM_001005289 | 1,8519 |

| | | |
|---|---|---|
| OR52K1 | NM_001005171 | 1,8497 |
| OR52K3P | AF143328 | 1,9580 |
| OR52N4 | NM_001005175 | 1,5723 |
| OSMR | NM_003999 | 2,2314 |
| P2RX3 | NM_002559 | -1,5369 |
| P2RY1 | NM_002563 | -1,5783 |
| PCDH19 | NM_001105243 | 1,5694 |
| PCSK6 | NM_002570 | -1,5008 |
| PDGFRL | NM_006207 | 3,0011 |
| PDIA5 | NM_006810 | 1,5859 |
| PDLIM1 | NM_020992 | 2,0447 |
| PHF11 | NM_001040443 | 2,6875 |
| PI16 | NM_153370 | -2,5010 |
| PIK3AP1 | NM_152309 | 1,5420 |
| PION | NM_017439 | 1,6291 |
| PITPNC1 | NM_181671 | -1,5158 |
| PLA2G4C | NM_003706 | 1,6217 |
| PLAT | NM_000930 | 2,7689 |
| PLCG2 | NM_002661 | 1,6606 |
| PLEKHA4 | NM_020904 | 1,8482 |
| PLEKHG1 | NM_001029884 | 1,7102 |
| PMAIP1 | NM_021127 | 1,7306 |
| PML | NM_033240 | 1,8133 |
| PMP22 | NM_000304 | 1,5970 |
| PNPT1 | NM_033109 | 1,9925 |
| PODXL | NM_001018111 | 2,1622 |
| POLR1E | NM_022490 | -1,5499 |
| POR | NM_000941 | 1,5852 |
| PPM1L | NM_139245 | -1,5612 |
| PREX2 | NM_024870 | 1,6567 |
| PRIC285 | NM_001037335 | 2,1344 |
| PRODH | NM_016335 | 2,2840 |
| PRSS23 | NM_007173 | 1,8940 |
| PSME2 | NM_002818 | 1,6438 |
| PSRC1 | NM_001032290 | 1,5542 |
| PTCHD1 | NM_173495 | -1,5377 |
| PTCHD2 | NM_020780 | -1,6445 |
| PTP4A3 | NM_032611 | 1,9207 |
| RAB27B | NM_004163 | -1,7997 |
| RAB3C | NM_138453 | 1,6733 |
| RARRES3 | NM_004585 | 4,5589 |
| RASGRP3 | NM_170672 | 3,0412 |
| REEP1 | NM_022912 | -2,0045 |
| REXO2 | NM_015523 | 1,5856 |
| RFC1 | L23320 | 1,6289 |
| RFTN1 | NM_015150 | 2,0738 |
| RGS6 | NM_004296 | 2,0430 |
| RGS9 | NM_003835 | -1,5893 |
| RIMS3 | NM_014747 | -1,5080 |
| RIPK1 | NM_003804 | 1,5615 |

| | | |
|---|---|---|
| RLBP1L2 | NM_001010852 | -1,9103 |
| RNF19B | NM_153341 | 1,5018 |
| RNF213 | NM_020914 | 0,3129 |
| ROBO3 | NM_022370 | -1,6036 |
| RPL27A | NM_000990 | -1,6283 |
| RPL7A | NM_000972 | -1,5421 |
| RPS3A | NM_001006 | -1,5209 |
| RTP4 | NM_022147 | 2,6960 |
| S100A10 | NM_002966 | 1,5932 |
| S100A11 | NM_005620 | 1,8356 |
| SAMD4A | NM_015589 | 1,5216 |
| SAMD9 | NM_017654 | 9,8080 |
| SCARNA12 | NR_003010 | -1,5065 |
| SCARNA9L | NR_023358 | -1,6145 |
| SCD | NM_005063 | -1,5565 |
| SCRT1 | NM_031309 | -1,5044 |
| SECTM1 | NM_003004 | 1,7327 |
| SEMA3D | NM_152754 | -2,0696 |
| SEMA4A | NM_022367 | -1,6181 |
| SERPINB8 | NM_002640 | 2,0605 |
| SERPINF1 | NM_002615 | 2,2505 |
| SERPING1 | NM_000062 | 3,4911 |
| SEZ6 | NM_178860 | 1,7186 |
| SFRP4 | NM_003014 | -1,5984 |
| SHISA5 | NM_016479 | 1,5712 |
| SLC12A7 | NM_006598 | 1,5405 |
| SLC15A3 | NM_016582 | 2,5991 |
| SLC17A8 | NM_139319 | -1,6421 |
| SLC1A4 | NM_003038 | 1,5369 |
| SLC34A2 | NM_006424 | 2,1493 |
| SLC39A10 | NM_001127257 | -1,5082 |
| SLC7A5 | NM_003486 | 1,7738 |
| SLCO4C1 | NM_180991 | 2,1962 |
| SLITRK6 | NM_032229 | -1,5957 |
| SLN | NM_003063 | 2,1985 |
| SMAD9 | NM_001127217 | -1,6208 |
| SNORA14A | NR_002955 | 1,8506 |
| SNORA21 | NR_002576 | -1,5332 |
| SNORA22 | NR_002961 | -1,5227 |
| SNORA24 | NR_002963 | -1,6165 |
| SNORA38B | NR_003706 | -1,5143 |
| SNORD113-3 | NR_003231 | 1,7589 |
| SOCS3 | NM_003955 | 2,4490 |
| SOD2 | NM_001024465 | 1,7607 |
| SP100 | NM_001080391 | 10,1203 |
| SP140L | NM_138402 | 3,9800 |
| SPARCL1 | NM_001128310 | 1,8868 |
| SPATS2L | NM_015535 | 1,5324 |
| SPRY4 | NM_030964 | 1,6814 |
| ST8SIA2 | NM_006011 | -1,6850 |

| | | |
|---|---|---|
| STMN2 | NM_007029 | -1,5543 |
| STX11 | NM_003764 | 1,5273 |
| SUSD1 | NM_022486 | 1,5245 |
| TAPBPL | NM_018009 | 2,1627 |
| TDRD7 | NM_014290 | 2,6342 |
| TEX2 | NM_018469 | 1,5043 |
| TFPI2 | NM_006528 | 2,5645 |
| THBS1 | NM_003246 | 5,2224 |
| TLR3 | NM_003265 | 3,3323 |
| TMEM132E | NM_207313 | 2,3596 |
| TMEM62 | NM_024956 | 1,6898 |
| TMOD1 | NM_003275 | -2,1331 |
| TNFAIP2 | NM_006291 | 3,2419 |
| TNFAIP3 | NM_006290 | 2,5329 |
| TNFSF10 | NM_003810 | 7,1207 |
| TNR | NM_003285 | -2,2778 |
| TNS3 | NM_022748 | 1,9086 |
| TNXB | NM_032470 | 1,6422 |
| TRIM21 | NM_003141 | 2,6781 |
| TRIM47 | NM_033452 | 1,5892 |
| TRIM5 | NM_033034 | 1,6882 |
| TSPAN18 | NM_130783 | -2,2196 |
| TSPAN7 | NM_004615 | -1,7920 |
| TUBB4 | NM_006087 | -1,6264 |
| UBQLNL | NM_145053 | 1,6896 |
| UNC93B1 | NM_030930 | 2,4500 |
| UNQ3104 | AY358109 | 1,5585 |
| VAMP5 | NM_006634 | 1,5124 |
| VCAM1 | NM_001078 | 1,9320 |
| VCAN | NM_004385 | -2,0749 |
| VEPH1 | NM_024621 | -1,7067 |
| WARS | NM_004184 | 2,7964 |
| WBSCR17 | NM_022479 | 1,7788 |
| ZCCHC18 | NM_001143978 | -1,5025 |
| ZCCHC2 | NM_017742 | 1,7911 |
| ZNFX1 | NM_021035 | 2,3118 |
