# Supplementary material for: ADAM23 promotes neuronal differentiation of human neural progenitor cells
Source: Cell Mol Biol Lett. 2017 Aug 18;22:16. doi: 10.1186/s11658-017-0045-1 (PMC5562998; doi:10.1186/s11658-017-0045-1)
Supplement: Supplementary file 5 — Enriched GO terms after downregulation of ADAM23. (PDF 271 kb) [file 11658_2017_45_MOESM5_ESM.pdf]

Significantly enriched GO terms after knockdown of ADAM23

	GOBPID	Pvalue	Count	Size	Term
1	GO:0060333	6,89E-01	19	83	interferon-gamma-mediated signaling pathway
2	GO:0034341	7,99E+00	23	149	response to interferon-gamma
3	GO:0009607	1,05E+03	51	846	response to biotic stimulus
4	GO:0051607	1,72E+05	26	305	defense response to virus
5	GO:0071345	2,88E+05	39	645	cellular response to cytokine stimulus
6	GO:0002684	3,95E+05	45	827	positive regulation of immune system process
7	GO:0002250	4,15E+05	26	318	adaptive immune response
8	GO:0051240	1,05E+07	56	1280	positive regulation of multicellular organismal process
9	GO:0060337	1,11E+07	12	80	type I interferon signaling pathway
10	GO:0034340	1,27E+07	12	81	response to type I interferon
11	GO:0001568	4,87E+07	31	540	blood vessel development
12	GO:0002376	5,20E+07	46	1144	immune system process
13	GO:0051707	5,99E+07	23	351	response to other organism
14	GO:0042221	6,60E+07	118	3847	response to chemical
15	GO:0009617	7,94E+06	28	468	response to bacterium
16	GO:0001765	9,56E+07	5	10	membrane raft assembly
17	GO:0006950	1,67E+08	70	2135	response to stress
18	GO:0052547	1,71E+08	24	377	regulation of peptidase activity
19	GO:0034138	2,17E+08	11	87	toll-like receptor 3 signaling pathway
20	GO:0022610	4,52E+08	53	1325	biological adhesion
21	GO:0045824	5,91E+08	7	34	negative regulation of innate immune response
22	GO:0002218	6,60E+07	19	275	activation of innate immune response
23	GO:0007166	8,05E+08	81	2437	cell surface receptor signaling pathway
24	GO:0019724	8,60E+08	11	100	B cell mediated immunity
25	GO:0043901	8,73E+08	13	140	negative regulation of multi-organism process
26	GO:0002443	9,16E+08	15	187	leukocyte mediated immunity
27	GO:0051247	1,11E+09	55	1439	positive regulation of protein metabolic process
28	GO:0051234	1,17E+09	125	4243	establishment of localization
29	GO:0045926	1,19E+09	16	212	negative regulation of growth
30	GO:0071294	1,49E+09	5	16	cellular response to zinc ion
31	GO:0051716	1,59E+09	174	6465	cellular response to stimulus
32	GO:0010038	1,79E+09	19	295	response to metal ion
33	GO:0030198	1,88E+09	20	327	extracellular matrix organization
34	GO:0002757	2,08E+09	24	437	immune response-activating signal transduction
35	GO:0014070	2,27E+09	35	780	response to organic cyclic compound
36	GO:0044700	2,78E+09	157	5736	single organism signaling
37	GO:0090132	2,86E+09	15	203	epithelium migration
38	GO:0031349	3,16E+08	22	393	positive regulation of defense response
39	GO:0072358	3,49E+09	37	863	cardiovascular system development
40	GO:0010562	3,64E+09	43	1070	positive regulation of phosphorus metabolic process
41	GO:0045765	4,57E+09	14	187	regulation of angiogenesis
42	GO:2000116	4,73E+09	15	212	regulation of cysteine-type endopeptidase activity
43	GO:0008219	5,32E+09	63	1823	cell death
44	GO:0043280	5,64E+09	11	122	positive regulation of cysteine-type endopeptidase activity involved in apoptotic process
45	GO:1990267	5,64E+09	11	122	response to transition metal nanoparticle
46	GO:0010950	5,68E+09	12	144	positive regulation of endopeptidase activity
47	GO:0042327	5,75E+09	39	952	positive regulation of phosphorylation
48	GO:0002221	6,28E+09	11	125	pattern recognition receptor signaling pathway
49	GO:0034142	6,54E+09	10	103	toll-like receptor 4 signaling pathway
50	GO:0051094	9,18E+09	41	1043	positive regulation of developmental process
51	GO:0065007	9,26E+08	249	10334	biological regulation
52	GO:2000026	0.000111327453614365	50	1395	regulation of multicellular organismal development
53	GO:0007568	0.000111532099063623	16	255	aging
54	GO:0001667	0.000116494397596559	17	283	ameboidal-type cell migration
55	GO:0006925	0.000121596722179128	4	13	inflammatory cell apoptotic process
56	GO:1902531	0.000122814983833186	53	1495	regulation of intracellular signal transduction
57	GO:0023051	0.000126016023783279	84	2714	regulation of signaling
58	GO:0006954	0.000128901226657932	22	444	inflammatory response
59	GO:1902041	0.000135284141212435	7	54	regulation of extrinsic apoptotic signaling pathway via death domain receptors
60	GO:0032268	0.000135715719660582	72	2236	regulation of cellular protein metabolic process
61	GO:0009605	0.000142338254740483	48	1458	response to external stimulus
62	GO:0071222	0.000150542906031221	11	136	cellular response to lipopolysaccharide
63	GO:0048002	0.000152743114407434	13	184	antigen processing and presentation of peptide antigen
64	GO:0031401	0.00015391631358429	42	1104	positive regulation of protein modification process
65	GO:0002504	0.000155246897486119	9	93	antigen processing and presentation of peptide or polysaccharide antigen via MHC class II
66	GO:0042771	0.000156209285927179	6	39	intrinsic apoptotic signaling pathway in response to DNA damage by p53 class mediator
67	GO:0034112	0.000170093484882946	13	186	positive regulation of homotypic cell-cell adhesion
68	GO:0002253	0.000179445137861761	6	42	activation of immune response
69	GO:0010811	0.000182776267501409	9	95	positive regulation of cell-substrate adhesion
70	GO:0032496	0.000186614795761154	11	143	response to lipopolysaccharide
71	GO:1903039	0.000189111894784019	13	188	positive regulation of leukocyte cell-cell adhesion
72	GO:0071241	0.000194508531872038	11	140	cellular response to inorganic substance
73	GO:0001932	0.000210517984504236	46	1266	regulation of protein phosphorylation
74	GO:0030335	0.000220159243633871	18	327	positive regulation of cell migration
75	GO:0042730	0.000227744042885156	5	27	fibrinolysis
76	GO:0010646	0.00023297175676565	84	2763	regulation of cell communication
77	GO:0030193	0.000286834701152778	8	80	regulation of blood coagulation
78	GO:0097202	0.000290808718065846	9	101	activation of cysteine-type endopeptidase activity

79	GO:0016485	0.000306034416360023	17	307	protein processing
80	GO:0032651	0.000309245210864457	6	44	regulation of interleukin-1 beta production
81	GO:0098602	0.00030961154160582	30	718	single organism cell adhesion
82	GO:0006911	0.000323588203328339	5	29	phagocytosis, engulfment
83	GO:0002526	0.000326824997132731	10	125	acute inflammatory response
84	GO:0032481	0.000340025635785948	8	82	positive regulation of type I interferon production
85	GO:0072593	0.00035383671337702	14	227	reactive oxygen species metabolic process
86	GO:0051051	0.000367981279664025	18	346	negative regulation of transport
87	GO:0044765	0.000391598735736134	94	3329	single-organism transport
88	GO:0040017	0.000391941102833823	18	345	positive regulation of locomotion
89	GO:0051272	0.000392233396317808	18	343	positive regulation of cellular component movement
90	GO:0010959	0.000397033971735087	17	314	regulation of metal ion transport
91	GO:0019220	0.000412850038147908	53	1570	regulation of phosphate metabolic process
92	GO:0045087	0.0004299110563357	22	527	innate immune response
93	GO:0002576	0.000434650707051191	8	85	platelet degranulation
94	GO:0044091	0.000447204500261569	5	31	membrane biogenesis
95	GO:0034124	0.000447622789278216	2	2	regulation of MyD88-dependent toll-like receptor signaling pathway
96	GO:0001817	0.000450591244817344	21	446	regulation of cytokine production
97	GO:0097305	0.000510551499383546	17	321	response to alcohol
98	GO:0006958	0.000520918835999434	5	32	complement activation, classical pathway
99	GO:1903531	0.000522322042901581	11	157	negative regulation of secretion by cell
100	GO:0050867	0.00054573192330676	15	265	positive regulation of cell activation
101	GO:0019886	0.000549622709652698	8	88	antigen processing and presentation of exogenous peptide antigen via MHC class II
102	GO:0031295	0.000575201000694714	7	68	T cell costimulation
103	GO:0034162	0.000575201000694714	7	68	toll-like receptor 9 signaling pathway
104	GO:0071498	0.000596135096705822	4	19	cellular response to fluid shear stress
105	GO:0071593	0.000599859902405648	20	417	lymphocyte aggregation
106	GO:0032735	0.000603403044477816	5	33	positive regulation of interleukin-12 production
107	GO:0009725	0.000610995326497062	37	996	response to hormone
108	GO:0042127	0.000614527094089683	30	778	regulation of cell proliferation
109	GO:0071260	0.000628719596835287	7	69	cellular response to mechanical stimulus
110	GO:0045785	0.000657086617310433	14	247	positive regulation of cell adhesion
111	GO:0043542	0.000715769250791249	10	138	endothelial cell migration
112	GO:0010529	0.000720078873330059	3	9	negative regulation of transposition
113	GO:0043627	0.000748659196575529	12	190	response to estrogen
114	GO:0050890	0.000755915600058104	14	245	cognition
115	GO:2001238	0.000773968640549416	6	52	positive regulation of extrinsic apoptotic signaling pathway
116	GO:0045595	0.000803094380648089	46	1346	regulation of cell differentiation
117	GO:0098609	0.000803994399937961	31	796	cell-cell adhesion
118	GO:0045861	0.000823386841740636	17	335	negative regulation of proteolysis
119	GO:0051259	0.000828428104307799	20	428	protein oligomerization
120	GO:0032760	0.000857467219784988	6	53	positive regulation of tumor necrosis factor production
121	GO:0048146	0.000857467219784988	6	53	positive regulation of fibroblast proliferation
122	GO:0035666	0.000883558212106793	7	73	TRIF-dependent toll-like receptor signaling pathway
123	GO:0045088	0.00100311493112614	17	348	regulation of innate immune response
124	GO:0032060	0.00101258678449661	3	10	bleb assembly
125	GO:0030224	0.00104651763102141	4	22	monocyte differentiation
126	GO:0006869	0.00106794650938793	15	283	lipid transport
127	GO:0010951	0.00108905580108711	13	226	negative regulation of endopeptidase activity
128	GO:0019884	0.00111258755996812	11	172	antigen processing and presentation of exogenous antigen
129	GO:0043123	0.00111258755996812	11	172	positive regulation of I-kappaB kinase/NF-kappaB signaling
130	GO:0051384	0.00116379700595927	10	147	response to glucocorticoid
131	GO:0030199	0.00117047808819457	5	38	collagen fibril organization
132	GO:0032570	0.00117047808819457	5	38	response to progesterone
133	GO:0044092	0.00118205861369364	36	996	negative regulation of molecular function
134	GO:0050891	0.00126285847750374	6	57	multicellular organismal water homeostasis
135	GO:0045862	0.00128739578347044	17	349	positive regulation of proteolysis
136	GO:0043067	0.00129293419642259	45	1339	regulation of programmed cell death
137	GO:0032879	0.00131694332904559	35	1018	regulation of localization
138	GO:0002457	0.00132401542243785	2	3	T cell antigen processing and presentation
139	GO:2000110	0.00132401542243785	2	3	negative regulation of macrophage apoptotic process
140	GO:0007160	0.00133454710894394	8	102	cell-matrix adhesion
141	GO:0001525	0.00134480114408092	12	210	angiogenesis
142	GO:0010632	0.00135613341390744	10	150	regulation of epithelial cell migration
143	GO:0070482	0.00136237708931765	15	290	response to oxygen levels
144	GO:0051251	0.0013806392698193	13	232	positive regulation of lymphocyte activation
145	GO:0009967	0.00146278799705206	43	1296	positive regulation of signal transduction
146	GO:0097300	0.00148236363563877	5	40	programmed necrotic cell death
147	GO:0071549	0.00150346196032733	4	24	cellular response to dexamethasone stimulus
148	GO:0030260	0.00151913885782953	7	80	entry into host cell
149	GO:0051828	0.00151913885782953	7	80	entry into other organism involved in symbiotic interaction
150	GO:0052126	0.00151913885782953	7	80	movement in host environment
151	GO:0010243	0.00155967572651599	33	902	response to organonitrogen compound
152	GO:0048870	0.00160899715322315	23	574	cell motility
153	GO:0061045	0.00165240718084041	6	60	negative regulation of wound healing
154	GO:0001666	0.00165888570174398	14	266	response to hypoxia
155	GO:0070372	0.00170190173176041	12	209	regulation of ERK1 and ERK2 cascade
156	GO:0002455	0.00172926644838788	3	12	humoral immune response mediated by circulating immunoglobulin
157	GO:0032944	0.00175180408719283	11	182	regulation of mononuclear cell proliferation
158	GO:0002544	0.00176027740155055	4	25	chronic inflammatory response
159	GO:0048534	0.00176104701925475	28	729	hematopoietic or lymphoid organ development

160	GO:0038166	0.00179885348288226	3	12	angiotensin-activated signaling pathway
161	GO:0044849	0.00179885348288226	3	12	estrous cycle
162	GO:0055022	0.00179885348288226	3	12	negative regulation of cardiac muscle tissue growth
163	GO:0032480	0.00185145601812192	5	42	negative regulation of type I interferon production
164	GO:0043410	0.00185487303711144	23	558	positive regulation of MAPK cascade
165	GO:2000145	0.00189139853568369	24	593	regulation of cell motility
166	GO:0022408	0.0018966443503908	9	131	negative regulation of cell-cell adhesion
167	GO:0048584	0.00200153592809429	13	267	positive regulation of response to stimulus
168	GO:0050776	0.002128610938167	21	527	regulation of immune response
169	GO:1903901	0.00220999977413617	6	64	negative regulation of viral life cycle
170	GO:0031998	0.0023020305110932	3	13	regulation of fatty acid beta-oxidation
171	GO:0045351	0.0023020305110932	3	13	type I interferon biosynthetic process
172	GO:0045651	0.0023020305110932	3	13	positive regulation of macrophage differentiation
173	GO:1901888	0.00230615508641567	6	64	regulation of cell junction assembly
174	GO:0048522	0.00241615040657653	93	3607	positive regulation of cellular process
175	GO:0060349	0.00246492523825133	7	87	bone morphogenesis
176	GO:0034614	0.00248531387425968	8	111	cellular response to reactive oxygen species
177	GO:0060341	0.00253867904894951	38	1117	regulation of cellular localization
178	GO:0008285	0.00256118689710043	24	607	negative regulation of cell proliferation
179	GO:0044763	0.00258023383388316	181	8747	single-organism cellular process
180	GO:0010716	0.0026109186384628	2	4	negative regulation of extracellular matrix disassembly
181	GO:0034136	0.0026109186384628	2	4	negative regulation of toll-like receptor 2 signaling pathway
182	GO:0034154	0.0026109186384628	2	4	toll-like receptor 7 signaling pathway
183	GO:0036018	0.0026109186384628	2	4	cellular response to erythropoietin
184	GO:1903365	0.0026109186384628	2	4	regulation of fear response
185	GO:2000987	0.0026109186384628	2	4	positive regulation of behavioral fear response
186	GO:2001181	0.0026109186384628	2	4	positive regulation of interleukin-10 secretion
187	GO:0051090	0.00265716413645944	16	342	regulation of sequence-specific DNA binding transcription factor activity
188	GO:0050878	0.00267127439547806	24	622	regulation of body fluid levels
189	GO:0030449	0.0027105035119358	4	28	regulation of complement activation
190	GO:0032689	0.0027105035119358	4	28	negative regulation of interferon-gamma production
191	GO:0050704	0.0027105035119358	4	28	regulation of interleukin-1 secretion
192	GO:1903792	0.0027105035119358	4	28	negative regulation of anion transport
193	GO:0051241	0.00279613762894489	30	843	negative regulation of multicellular organismal process
194	GO:0002521	0.00280703175354848	15	318	leukocyte differentiation
195	GO:0044708	0.00296851651431465	17	381	single-organism behavior
196	GO:0071384	0.00306341698767273	5	47	cellular response to corticosteroid stimulus
197	GO:2001267	0.003092409961313	4	29	regulation of cysteine-type endopeptidase activity involved in apoptotic signaling pathway
198	GO:0060401	0.00326644643914452	8	116	cytosolic calcium ion transport
199	GO:0002456	0.00327665114097848	6	69	T cell mediated immunity
200	GO:0002753	0.00336139883647569	5	48	cytoplasmic pattern recognition receptor signaling pathway
201	GO:1900047	0.00336139883647569	5	48	negative regulation of hemostasis
202	GO:0046627	0.00350945283502664	4	30	negative regulation of insulin receptor signaling pathway
203	GO:0022898	0.00354611609953671	10	171	regulation of transmembrane transporter activity
204	GO:1902187	0.00354913243040216	3	15	negative regulation of viral release from host cell
205	GO:0007613	0.00358974995186123	7	93	memory
206	GO:0032846	0.00359550593065083	9	144	positive regulation of homeostatic process
207	GO:0009612	0.00361171484346098	8	120	response to mechanical stimulus
208	GO:0032101	0.00367569288412435	19	471	regulation of response to external stimulus
209	GO:0043549	0.00373840853132161	30	842	regulation of kinase activity
210	GO:0051092	0.00381925490209303	8	119	positive regulation of NF-kappaB transcription factor activity
211	GO:0000165	0.0038913003957463	12	240	MAPK cascade
212	GO:0001961	0.00396311925499184	4	31	positive regulation of cytokine-mediated signaling pathway
213	GO:0080134	0.00397346276583875	32	972	regulation of response to stress
214	GO:0010332	0.00401897745238002	5	50	response to gamma radiation
215	GO:0050819	0.00401897745238002	5	50	negative regulation of coagulation
216	GO:0072376	0.00417527681780875	6	72	protein activation cascade
217	GO:0071805	0.00417747052988901	10	175	potassium ion transmembrane transport
218	GO:0042060	0.00420332830380299	24	639	wound healing
219	GO:0048878	0.00421707210648729	33	961	chemical homeostasis
220	GO:0002158	0.00429065029450463	2	5	osteoclast proliferation
221	GO:0022614	0.00429065029450463	2	5	membrane to membrane docking
222	GO:0031340	0.00429065029450463	2	5	positive regulation of vesicle fusion
223	GO:0033034	0.00429065029450463	2	5	positive regulation of myeloid cell apoptotic process
224	GO:0045657	0.00429065029450463	2	5	positive regulation of monocyte differentiation
225	GO:0071280	0.00429065029450463	2	5	cellular response to copper ion
226	GO:0071276	0.00430024030116127	3	16	cellular response to cadmium ion
227	GO:0050792	0.00434839825079454	10	176	regulation of viral process
228	GO:0042391	0.00435147507909921	15	331	regulation of membrane potential
229	GO:0050663	0.00444031516482435	8	123	cytokine secretion
230	GO:1902107	0.00444175291628897	8	122	positive regulation of leukocyte differentiation
231	GO:0032413	0.00476370203556185	5	52	negative regulation of ion transmembrane transporter activity
232	GO:0032940	0.00486837562478022	26	729	secretion by cell
233	GO:0002209	0.00498609791371668	4	33	behavioral defense response
234	GO:0032732	0.00498609791371668	4	33	positive regulation of interleukin-1 production
235	GO:0030099	0.00512466163730392	14	304	myeloid cell differentiation
236	GO:0001977	0.0051406125976744	3	17	renal system process involved in regulation of blood volume
237	GO:0002861	0.0051406125976744	3	17	regulation of inflammatory response to antigenic stimulus
238	GO:0031112	0.0051406125976744	3	17	positive regulation of microtubule polymerization or depolymerization
239	GO:0032703	0.0051406125976744	3	17	negative regulation of interleukin-2 production
240	GO:0006898	0.00523773599924395	12	240	receptor-mediated endocytosis

241	GO:0019932	0.00543309449832059	11	211	second-messenger-mediated signaling
242	GO:0033555	0.00544656864389586	6	76	multicellular organismal response to stress
243	GO:1903317	0.00544656864389586	6	76	regulation of protein maturation
244	GO:2000106	0.00544656864389586	6	76	regulation of leukocyte apoptotic process
245	GO:0045843	0.00555820396600721	4	34	negative regulation of striated muscle tissue development
246	GO:0071622	0.00555820396600721	4	34	regulation of granulocyte chemotaxis
247	GO:0086009	0.00555820396600721	4	34	membrane repolarization
248	GO:1903034	0.00559074999542269	11	218	regulation of response to wounding
249	GO:0045669	0.00560150571910311	5	54	positive regulation of osteoblast differentiation
250	GO:0001776	0.00580428198942263	6	77	leukocyte homeostasis
251	GO:0034763	0.00580428198942263	6	77	negative regulation of transmembrane transport
252	GO:0045860	0.00582623187151969	22	577	positive regulation of protein kinase activity
253	GO:0060135	0.00605714140525609	5	55	maternal process involved in female pregnancy
254	GO:0030220	0.00607302111687732	3	18	platelet formation
255	GO:0032607	0.00607302111687732	3	18	interferon-alpha production
256	GO:0034123	0.00607302111687732	3	18	positive regulation of toll-like receptor signaling pathway
257	GO:0035455	0.00607302111687732	3	18	response to interferon-alpha
258	GO:0072606	0.00607302111687732	3	18	interleukin-8 secretion
259	GO:1901881	0.00607302111687732	3	18	positive regulation of protein depolymerization
260	GO:2000279	0.00607302111687732	3	18	negative regulation of DNA biosynthetic process
261	GO:0045216	0.00623493158036715	11	215	cell-cell junction organization
262	GO:0002578	0.00634608923168721	2	6	negative regulation of antigen processing and presentation
263	GO:0002866	0.00634608923168721	2	6	positive regulation of acute inflammatory response to antigenic stimulus
264	GO:0003097	0.00634608923168721	2	6	renal water transport
265	GO:0045343	0.00634608923168721	2	6	regulation of MHC class I biosynthetic process
266	GO:0045354	0.00634608923168721	2	6	regulation of interferon-alpha biosynthetic process
267	GO:0045869	0.00634608923168721	2	6	negative regulation of single stranded viral RNA replication via double stranded DNA intermediate
268	GO:0046877	0.00634608923168721	2	6	regulation of saliva secretion
269	GO:0070383	0.00634608923168721	2	6	DNA cytosine deamination
270	GO:0097527	0.00634608923168721	2	6	necroptotic signaling pathway
271	GO:0003008	0.00636077932397246	55	1861	system process
272	GO:0051346	0.00642298794321779	16	375	negative regulation of hydrolase activity
273	GO:0043085	0.00652813569660333	44	1419	positive regulation of catalytic activity
274	GO:0032677	0.00653822754721617	5	56	regulation of interleukin-8 production
275	GO:1904063	0.00653822754721617	5	56	negative regulation of cation transmembrane transport
276	GO:0006812	0.00655222927654272	33	990	cation transport
277	GO:0001909	0.0065706943618272	6	79	leukocyte mediated cytotoxicity
278	GO:0002690	0.0065706943618272	6	79	positive regulation of leukocyte chemotaxis
279	GO:0007044	0.0065706943618272	6	79	cell-substrate junction assembly
280	GO:0007268	0.00677411060216798	25	693	synaptic transmission
281	GO:0043270	0.00689514804144082	11	218	positive regulation of ion transport
282	GO:0008015	0.00697246467841166	17	412	blood circulation
283	GO:0032729	0.00704547457315006	5	57	positive regulation of interferon-gamma production
284	GO:0060395	0.00704547457315006	5	57	SMAD protein signal transduction
285	GO:0010818	0.00709993724565949	3	19	T cell chemotaxis
286	GO:0097205	0.00709993724565949	3	19	renal filtration
287	GO:0043269	0.00720778711300769	4	38	regulation of ion transport
288	GO:0045321	0.00754388703347712	20	528	leukocyte activation
289	GO:0010522	0.00785441285812679	6	82	regulation of calcium ion transport into cytosol
290	GO:0050852	0.00808788218094621	7	108	T cell receptor signaling pathway
291	GO:0098661	0.00808788218094621	7	108	inorganic anion transmembrane transport
292	GO:0035456	0.00822354635166561	3	20	response to interferon-beta
293	GO:0050718	0.00822354635166561	3	20	positive regulation of interleukin-1 beta secretion
294	GO:0060716	0.00822354635166561	3	20	labyrinthine layer blood vessel development
295	GO:0097296	0.00822354635166561	3	20	activation of cysteine-type endopeptidase activity involved in apoptotic signaling pathway
296	GO:0032653	0.00828141544368885	4	38	regulation of interleukin-10 production
297	GO:0071219	0.00832807162677985	2	7	cellular response to molecule of bacterial origin
298	GO:0032103	0.00834371849115987	12	255	positive regulation of response to external stimulus
299	GO:0042129	0.0084341582207462	8	136	regulation of T cell proliferation
300	GO:0019882	0.00850021811305833	3	21	antigen processing and presentation
301	GO:0071310	0.00852517890328257	41	1456	cellular response to organic substance
302	GO:0045670	0.00873113959642851	5	60	regulation of osteoclast differentiation
303	GO:0061035	0.00873113959642851	5	60	regulation of cartilage development
304	GO:0002606	0.00876065956214721	2	7	positive regulation of dendritic cell antigen processing and presentation
305	GO:0033632	0.00876065956214721	2	7	regulation of cell-cell adhesion mediated by integrin
306	GO:0046598	0.00876065956214721	2	7	positive regulation of viral entry into host cell
307	GO:0048251	0.00876065956214721	2	7	elastic fiber assembly
308	GO:0051549	0.00876065956214721	2	7	positive regulation of keratinocyte migration
309	GO:0060406	0.00876065956214721	2	7	positive regulation of penile erection
310	GO:0002695	0.00879751639426887	8	137	negative regulation of leukocyte activation
311	GO:0007229	0.00880473749838374	6	84	integrin-mediated signaling pathway
312	GO:0008344	0.00880473749838374	6	84	adult locomotory behavior
313	GO:0034765	0.00890838403406401	15	355	regulation of ion transmembrane transport
314	GO:0061448	0.00892502132494115	11	226	connective tissue development
315	GO:0016192	0.00897834501341111	38	1207	vesicle-mediated transport
316	GO:1903556	0.00907704746411059	4	39	negative regulation of tumor necrosis factor superfamily cytokine production
317	GO:0002697	0.00915207842722177	15	360	regulation of immune effector process
318	GO:1903035	0.00917054712140082	6	86	negative regulation of response to wounding
319	GO:0002685	0.00917245758891936	8	138	regulation of leukocyte migration
320	GO:0048513	0.00929389356791594	59	2158	organ development
321	GO:0001952	0.00930955156794256	6	85	regulation of cell-matrix adhesion

322	GO:0019229	0.00934993482940189	5	61	regulation of vasoconstriction
323	GO:0033692	0.00934993482940189	5	61	cellular polysaccharide biosynthetic process
324	GO:0034146	0.00934993482940189	5	61	toll-like receptor 5 signaling pathway
325	GO:0003416	0.00944576164130158	3	21	endochondral bone growth
326	GO:0006817	0.00944576164130158	3	21	phosphate ion transport
327	GO:0019430	0.00944576164130158	3	21	removal of superoxide radicals
328	GO:0035640	0.00944576164130158	3	21	exploration behavior
329	GO:0046621	0.00944576164130158	3	21	negative regulation of organ growth
330	GO:0060713	0.00944576164130158	3	21	labyrinthine layer morphogenesis
331	GO:0015711	0.0096151009604945	16	392	organic anion transport
332	GO:0042110	0.00980647537353564	14	330	T cell activation
333	GO:0050868	0.00983470243287504	6	86	negative regulation of T cell activation
334	GO:0009653	0.00986499102200072	71	2575	anatomical structure morphogenesis
335	GO:0031032	0.00995795993528057	8	140	actomyosin structure organization
