# Supplementary figures and images for: ADAM23 promotes neuronal differentiation of human neural progenitor cells
Source: Cell Mol Biol Lett. 2017 Aug 18;22:16. doi: 10.1186/s11658-017-0045-1 (PMC5562998; doi:10.1186/s11658-017-0045-1)

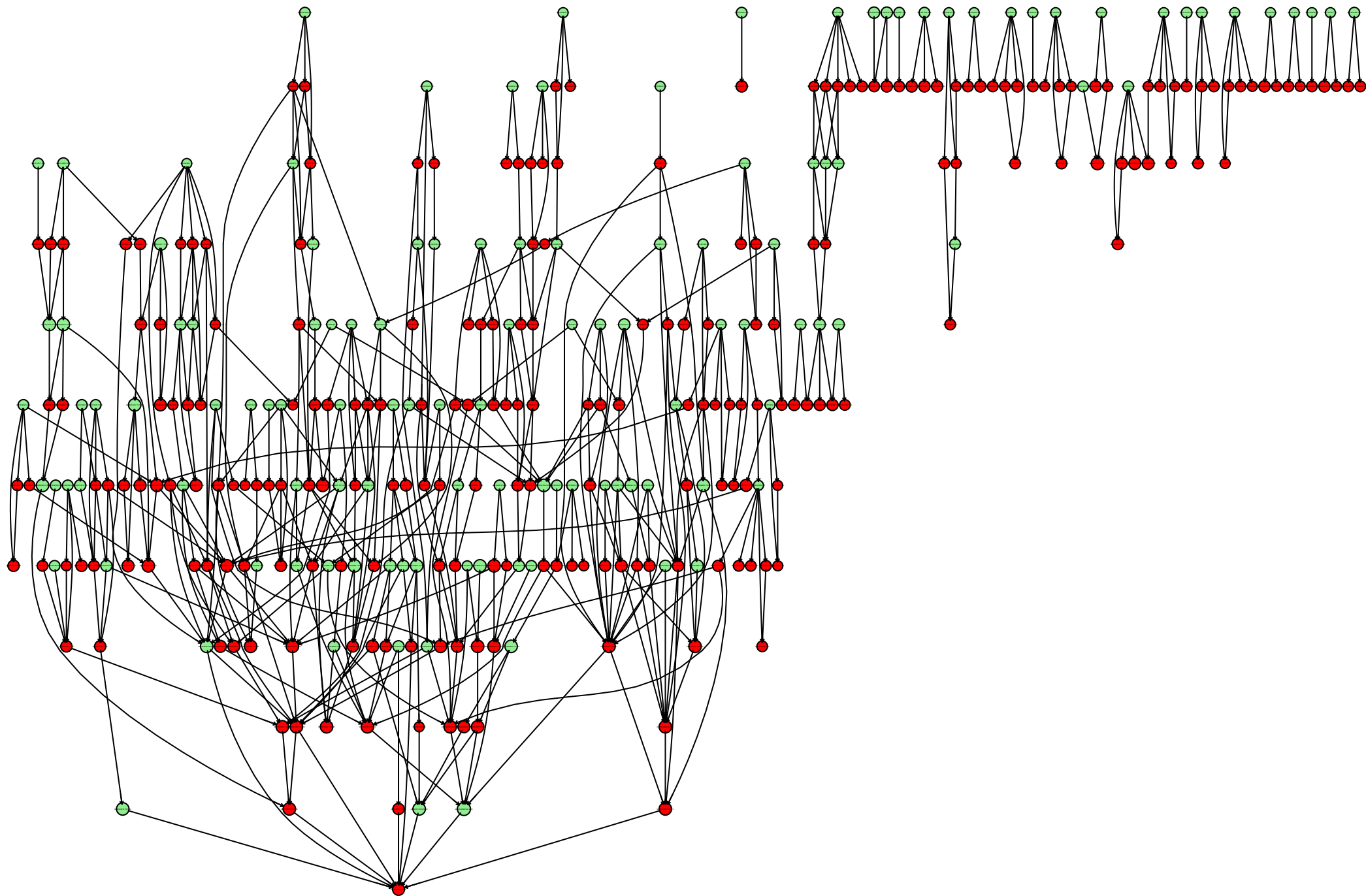

Supplement: Supplementary file 7 — The GO tree for significantly enriched GO terms after overexpression of ADAM23. Significantly enriched GO terms (yellow circles) after overexpression of ADAM23 are illustrated in a GO tree together with the required connective GO terms (red circles). All GO terms are represented together with their corresponding daughter GO terms. Twelve generations of GO terms are included. The GO term at the bottom presents the first-generation GO term (molecular function). (PDF 87 kb) [file 11658_2017_45_MOESM7_ESM.pdf]
